# Supplementary material for: Synthesis of New 2,3-Dihydroindole Derivatives and Evaluation of Their Melatonin Receptor Binding Affinity
Source: Molecules. 2022 Nov 2;27(21):7462. doi: 10.3390/molecules27217462 (PMC9655341; doi:10.3390/molecules27217462)

Supplementary Materials

Synthesis of New 2,3-Dihydroindole Derivatives and Evaluation of Its Melatonin Receptor Binding Affinity

S1. Binding curves of compounds 7a and 7d to MT2 (A,B) and MT1 (C)

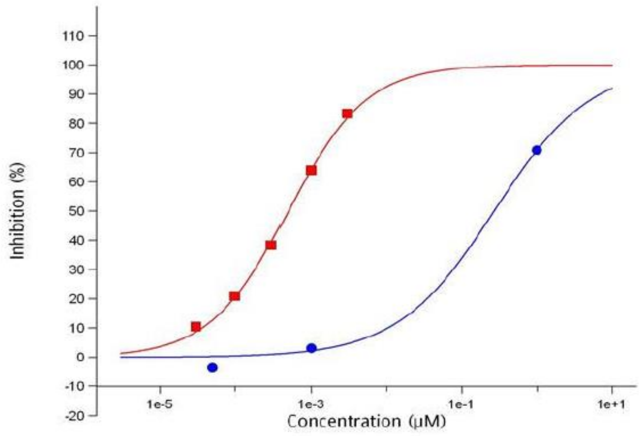

Compound	IC_{50}	K_i	n_H
7a	0.268 µM	0.139 µM	0.676
4-P-PDOT	0.497 nM	0.258 nM	0.840

Figure A

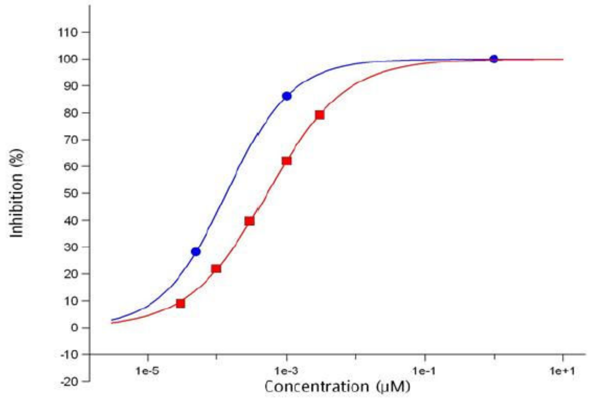

Compound	IC_{50}	K_i	n_H
7d	0.138 nM	0.0716 nM	0.925
Melatonin	0.527 nM	0.274 nM	0.774

Figure B

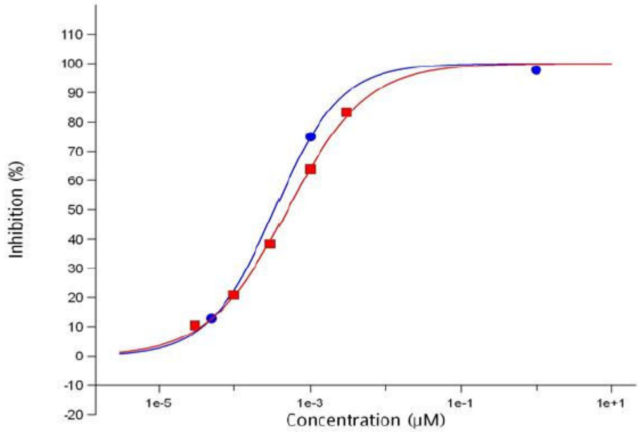

Compound	IC_{50}	K_i	n_H
7d	0.338 nM	0.176 nM	1.01
4-P-PDOT	0.497 nM	0.258 nM	0.840

Figure C

METHODS - RADIOLIGAND BINDING ASSAYS

■ 251600 Melatonin MT_1

Source:	Human recombinant CHO-K1 cells
Ligand:	0.05 nM [^{125}I] 2-Iodomelatonin
Vehicle:	1% DMSO
Incubation Time/Temp:	3 hours @ 25ºC
Incubation Buffer:	25 mM HEPES, pH 7.4, 5 mM $MgCl_2$, 1 mM $CaCl_2$, 0.5% BSA
Non-Specific Ligand:	1 μM 6-Chloromelatonin
K_D:	0.054 nM *
B_{MAX}:	3.5 pmole/mg Protein *
Specific Binding:	97% *
Quantitation Method:	Radioligand Binding
Significance Criteria:	≥ 50% of max stimulation or inhibition

■ 251700 Melatonin MT_2

Source:	Human recombinant CHO-K1 cells
Ligand:	0.05 nM [^{125}I] 2-Iodomelatonin
Vehicle:	1% DMSO
Incubation Time/Temp:	4 hours @ 25ºC
Incubation Buffer:	25 mM HEPES, pH 7.4, 5 mM $MgCl_2$, 1 mM $CaCl_2$, 0.1% BSA
Non-Specific Ligand:	1 μM 6-Chloromelatonin
K_D:	0.054 nM *
B_{MAX}:	0.93 pmole/mg Protein *
Specific Binding:	98% *
Quantitation Method:	Radioligand Binding
Significance Criteria:	≥ 50% of max stimulation or inhibition

Selected NMR spectra of synthesized compounds

S2. 1H NMR spectrum of cyano(*N*-methyl-2-oxo-5-methoxy indolin-3-ylidene)acetic acid (**2g**)

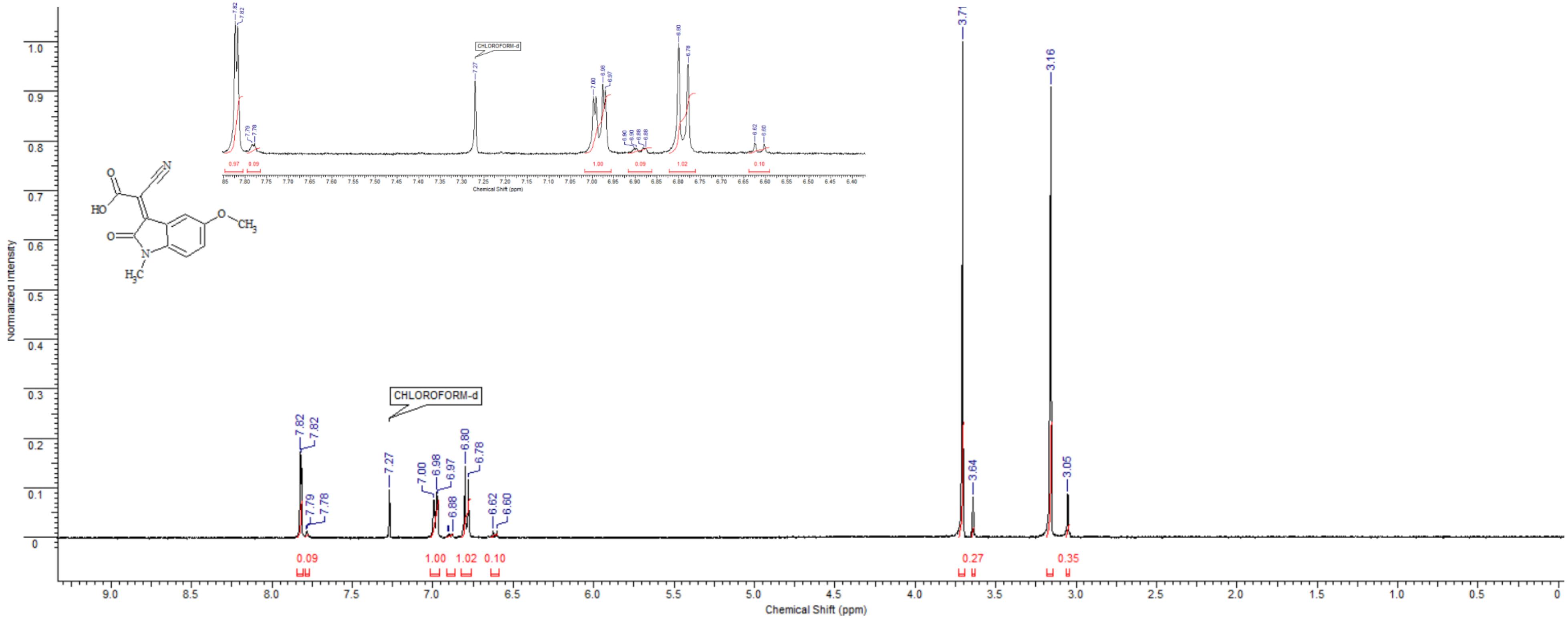

S3. 13C NMR spectrum of cyano(*N*-methyl-2-oxo-5-methoxy indolin-3-ylidene)acetic acid (**2g**)

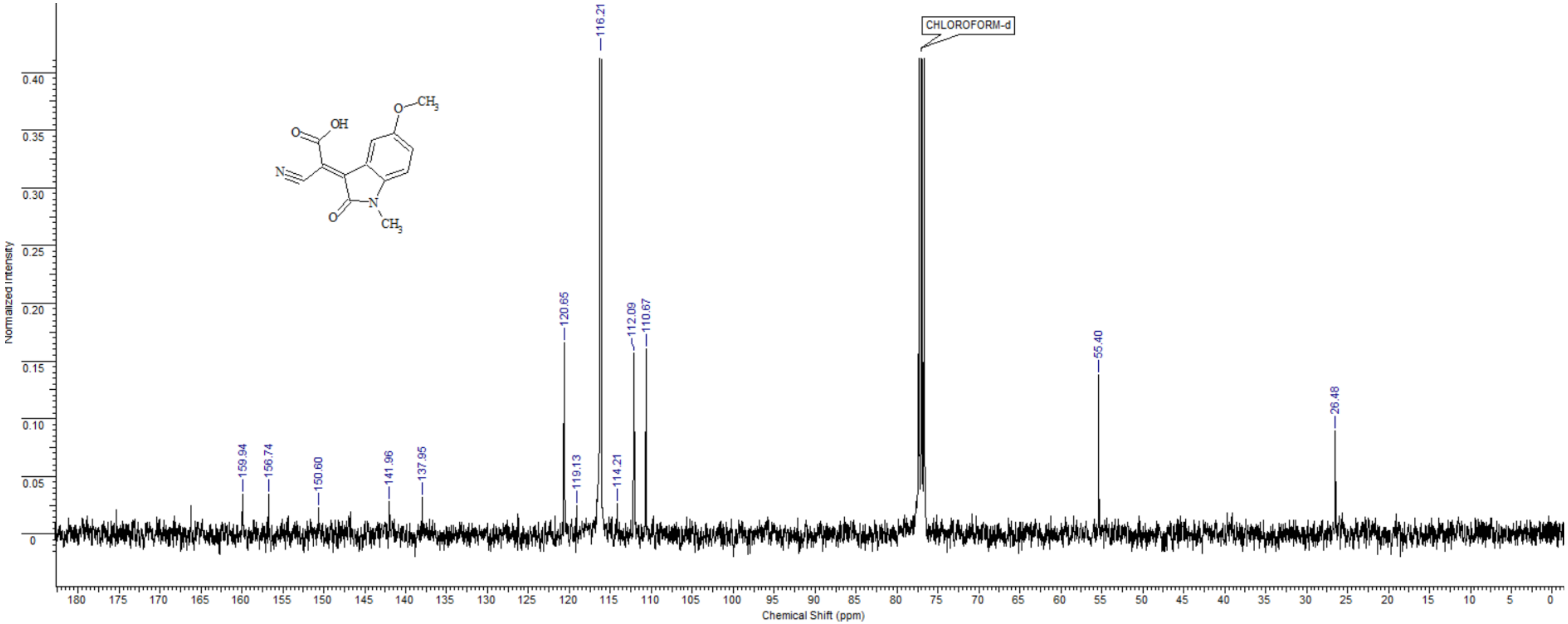

S4. 1H NMR spectrum of 2-(5-Bromo-1-methyl-2-oxo-2,3- dihydro-1H-indol-3-ylidene)acetonitrile (**3i**)

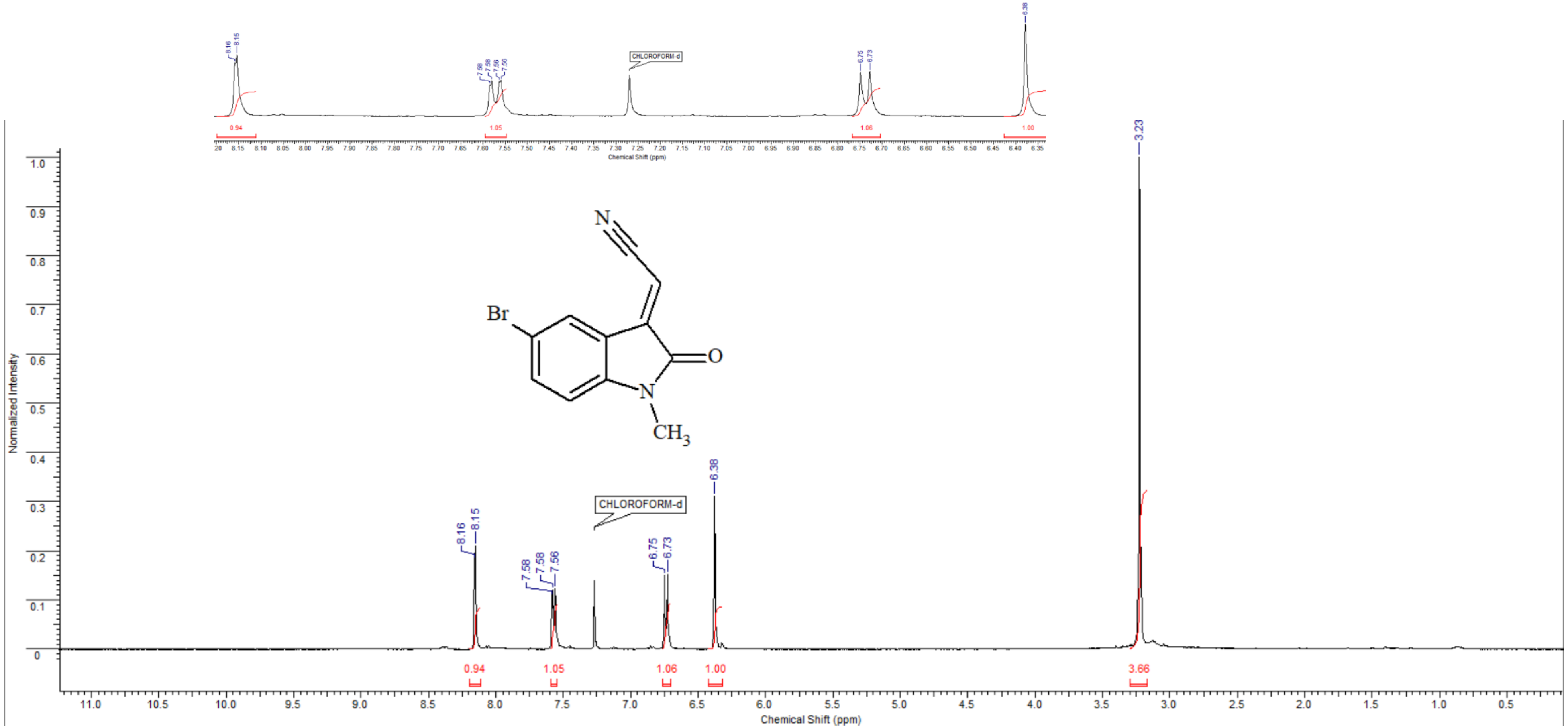

S5 1H NMR spectrum of 2-(1,6,7-Trimethyl-2-oxo-2,3-dihydro-*1H*-indol-3-ylidene)acetonitrile (**3j**)

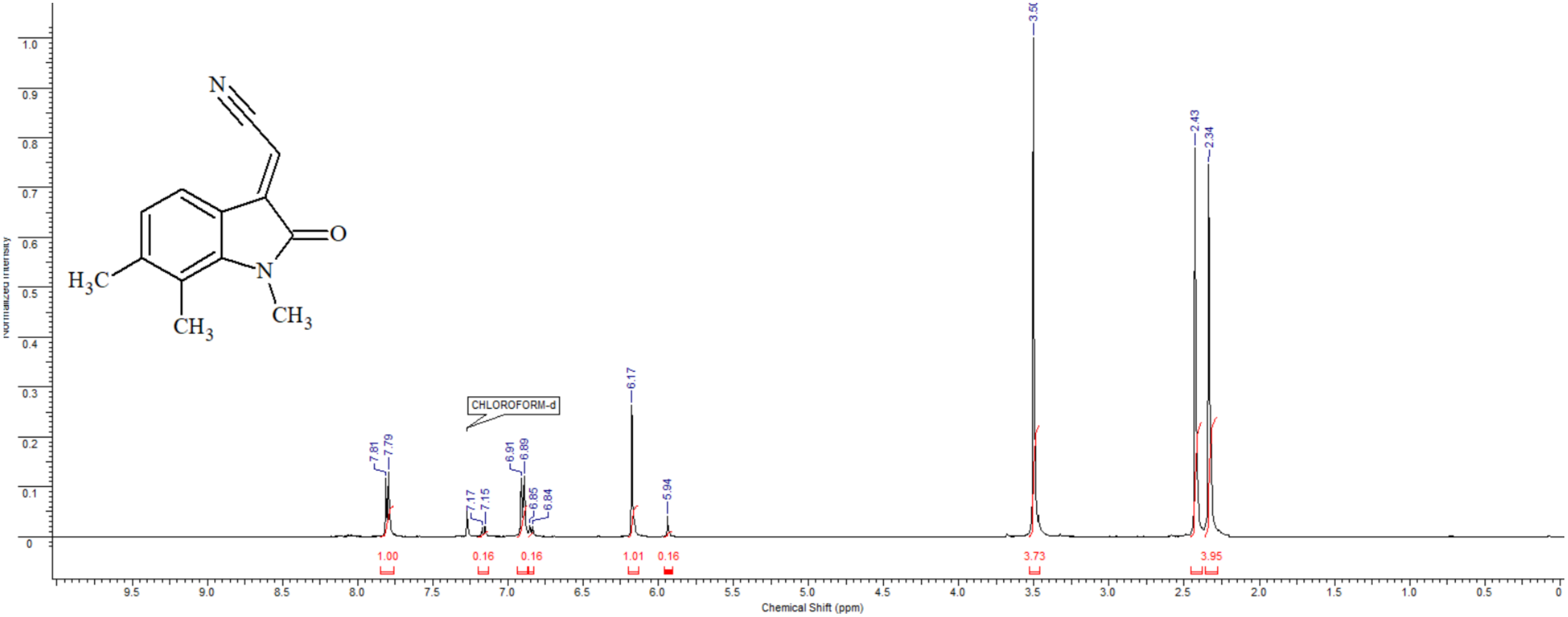

S6 13C NMR spectrum of 2-(1,6,7-Trimethyl-2-oxo-2,3-dihydro-*1H*-indol-3-ylidene)acetonitrile (**3j**)

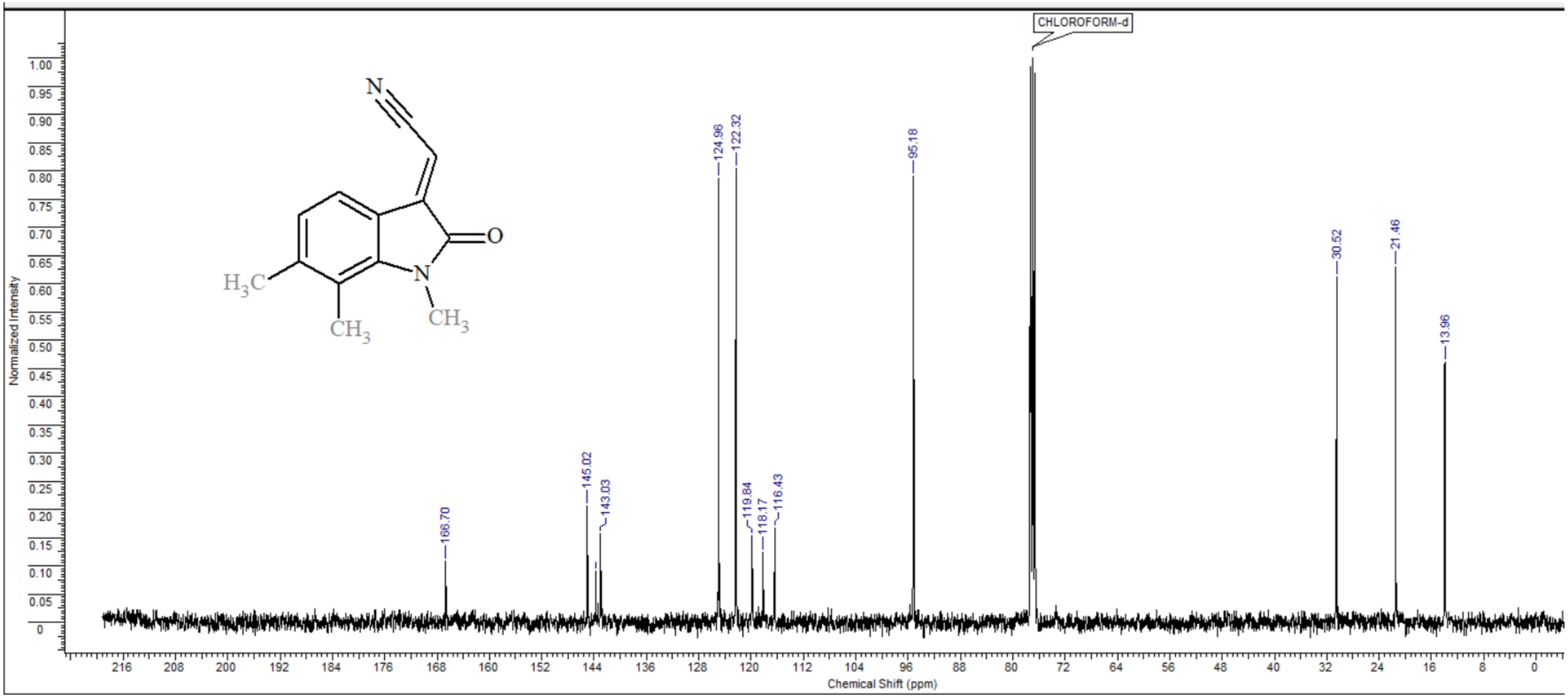

S7 1H NMR spectrum of 2-(1-methyl-5-methoxy-2-oxo-2,3-dihydro-1*H*-indol-3-yl)acetonitrile (**4g**)

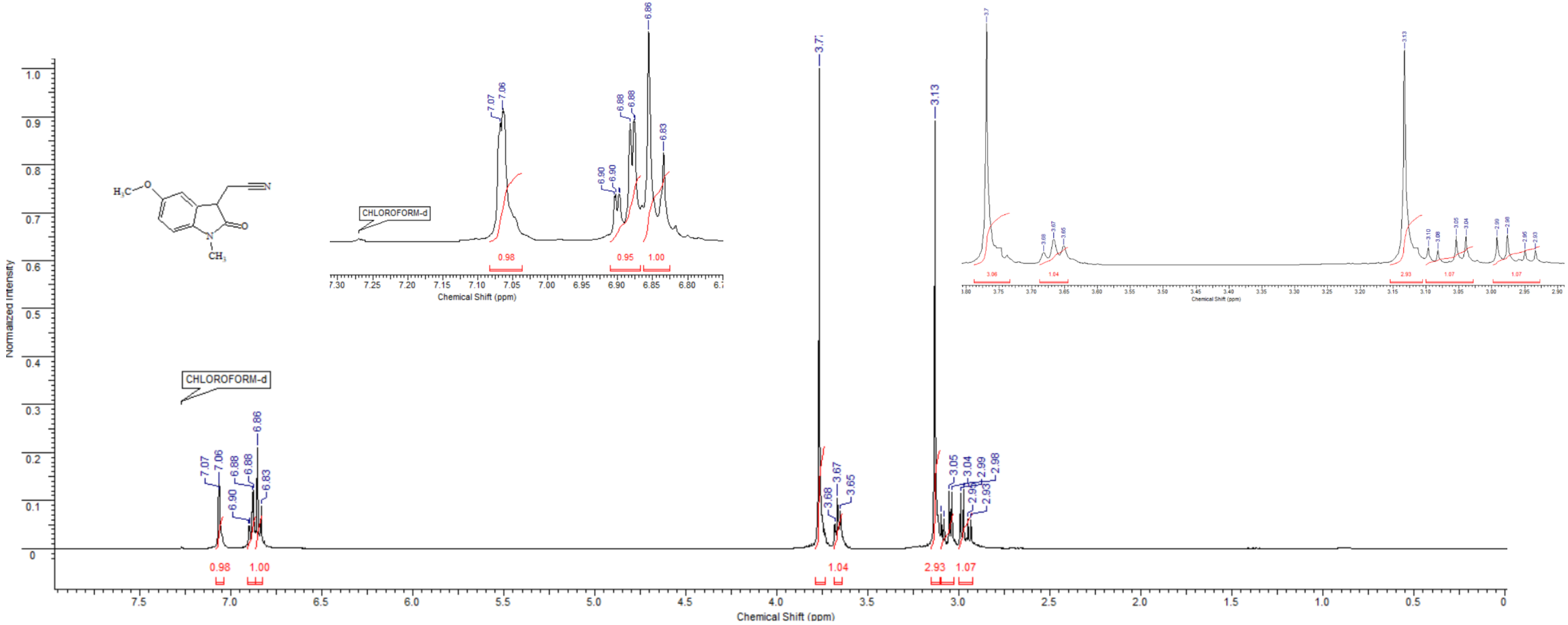
Normalized Intensity
Chemical Shift (ppm)
CHLOROFORM-d

S8 1H NMR spectrum of 2-(5-bromo-1-methyl-2-oxo-2,3-dihydro-1*H*-indol-3-yl)acetonitrile (**4i**)

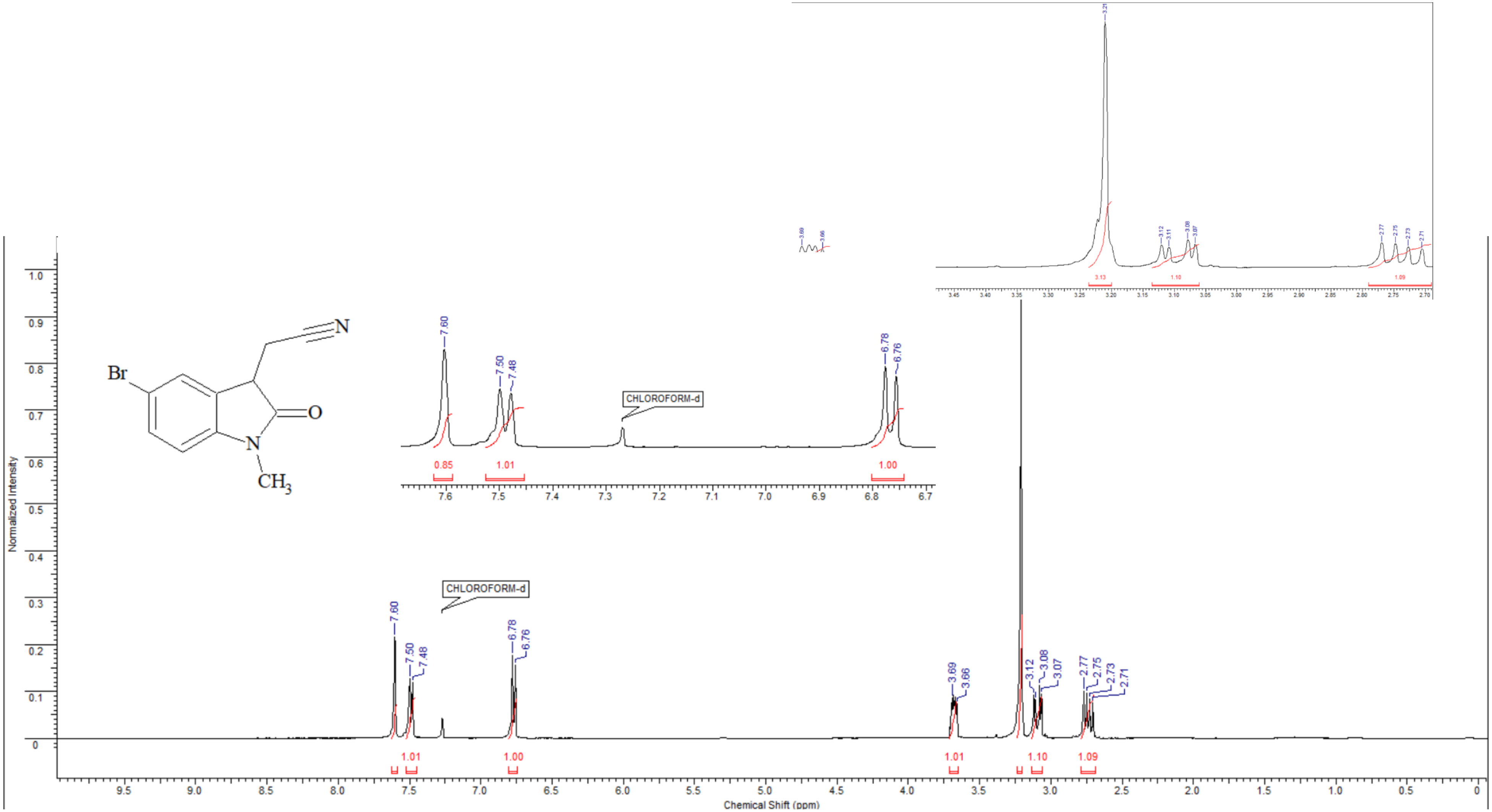

S9 13C NMR spectrum of 2-(5-Bromo-1-methyl-2-oxo-2,3-dihydro-1H-indol-3-yl)acetonitrile (**4i**)

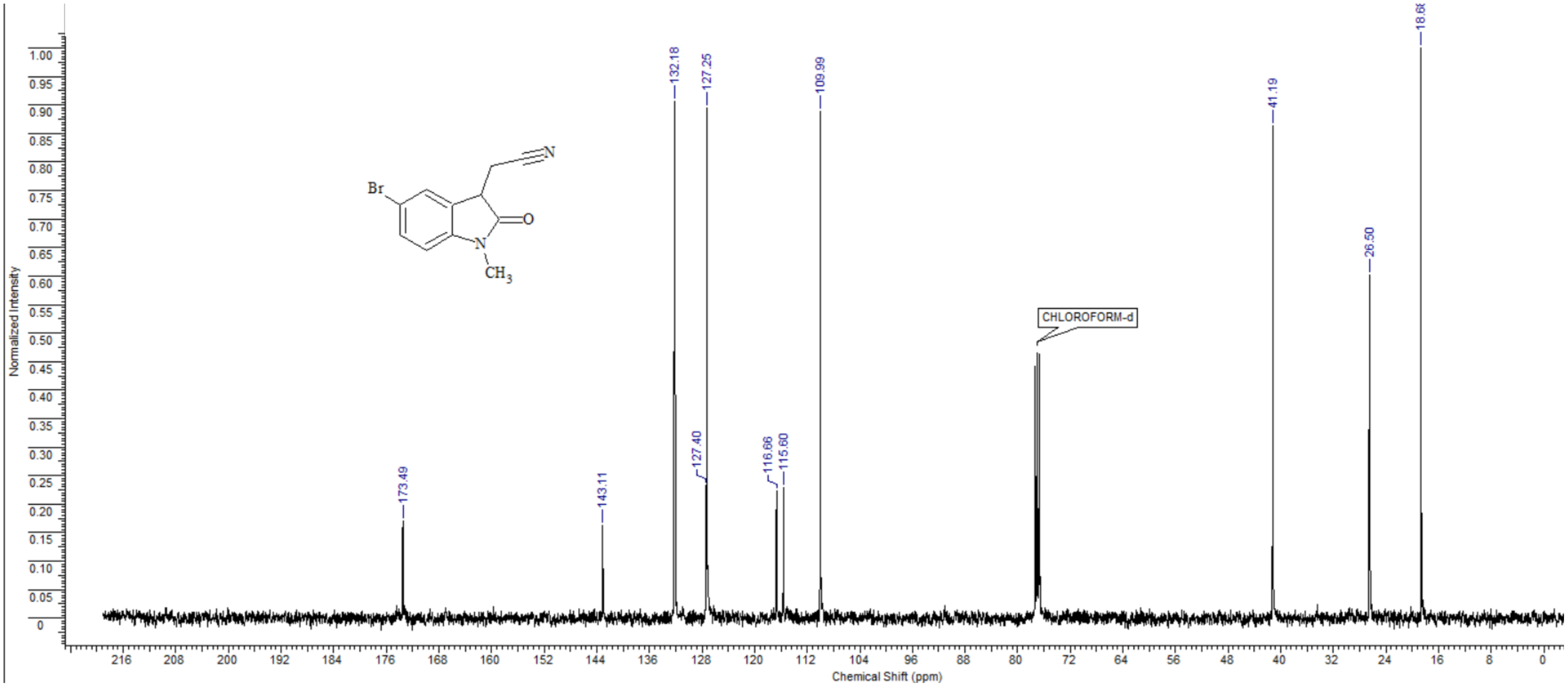

S10 1H NMR spectrum of Tert-butyl 3-(cyanomethyl)-2-oxo-2,3-dihydro-*1H*-indol-1-carboxylate (**4k**)

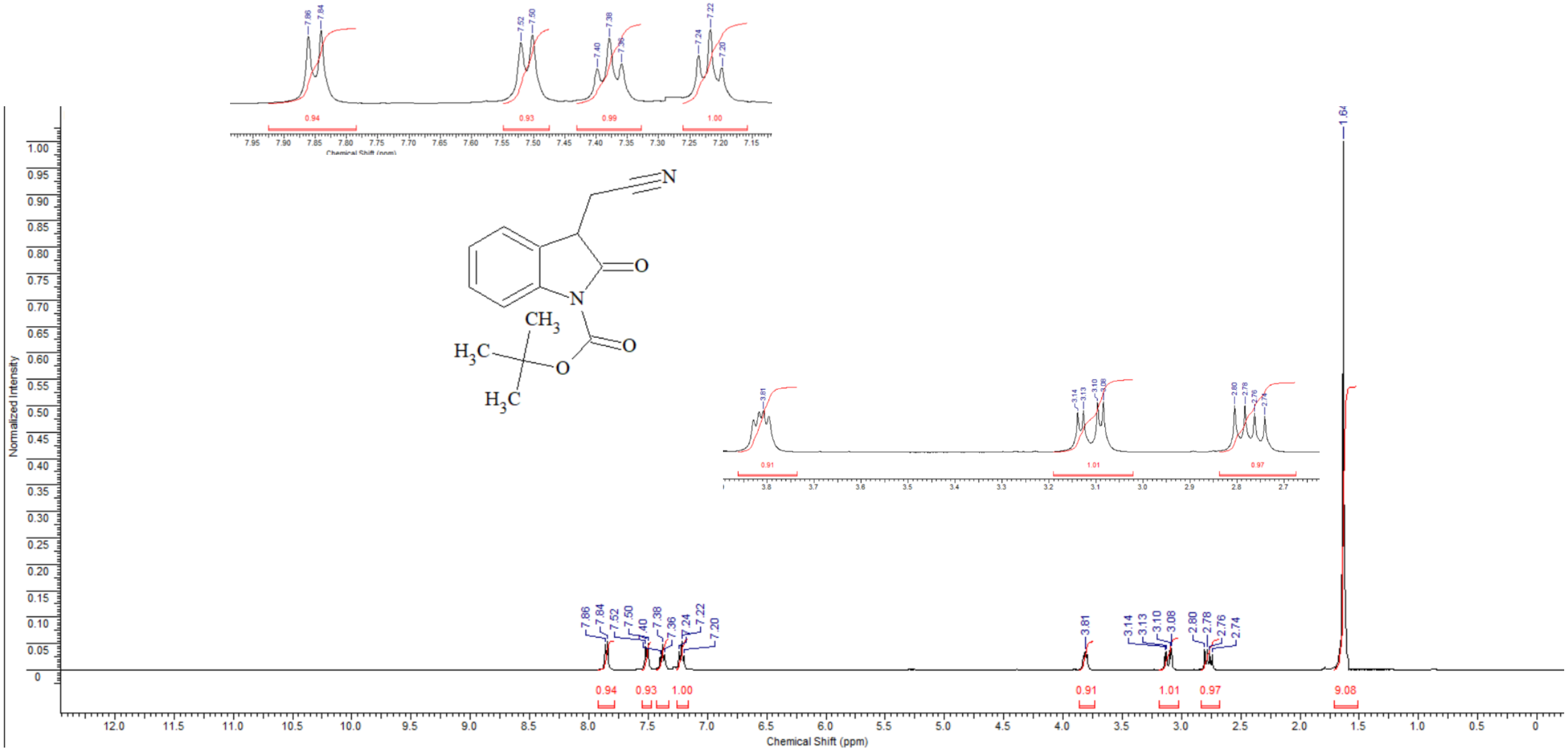

S11 13C NMR spectrum of Tert-butyl 3-(cyanomethyl)-2-oxo-2,3-dihydro-*1H*-indol-1-carboxylate (**4k**)

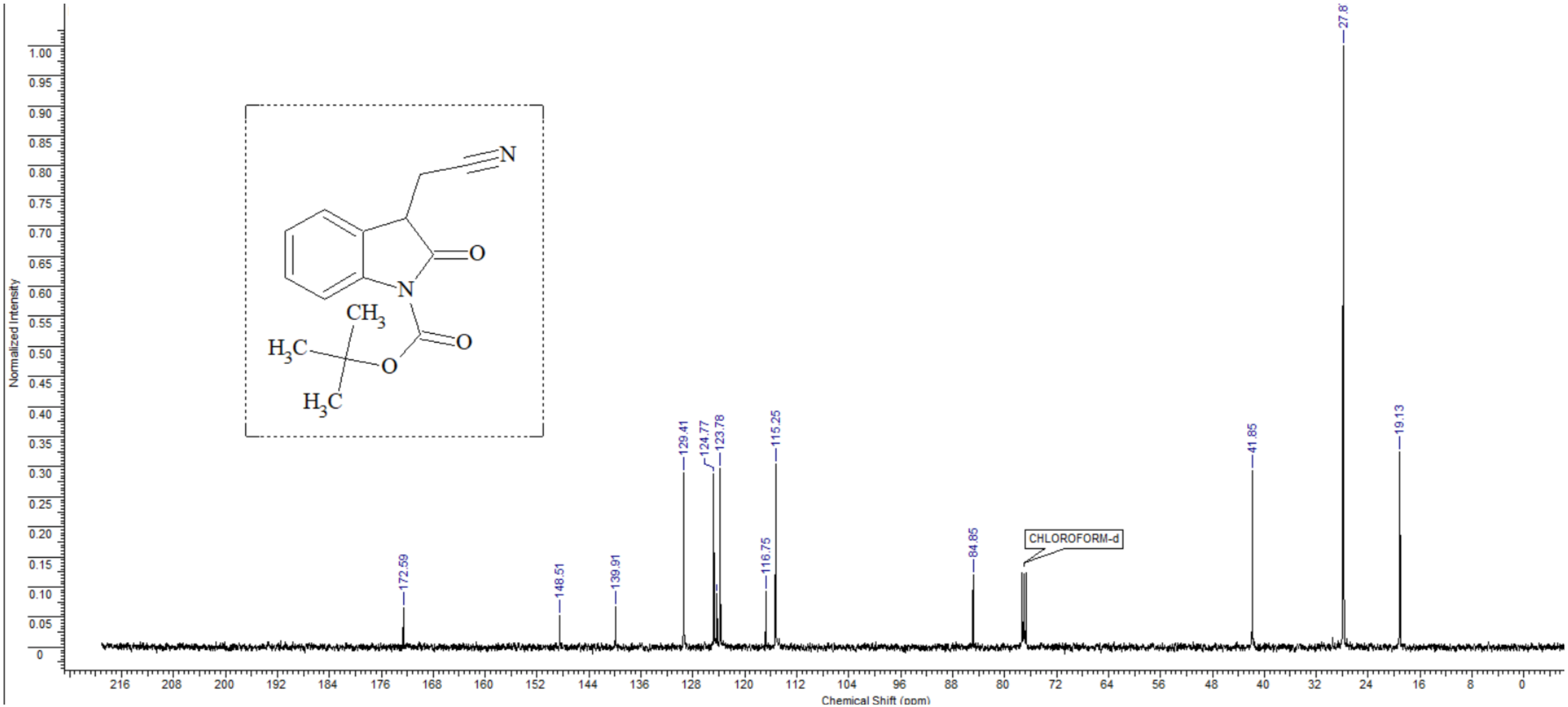

S12 1H NMR spectrum of Tert-butyl 5-methoxy-3-(cyanomethyl)-2-oxo-2,3-dihydro-1H-indol-1-carboxylate (**4l**)

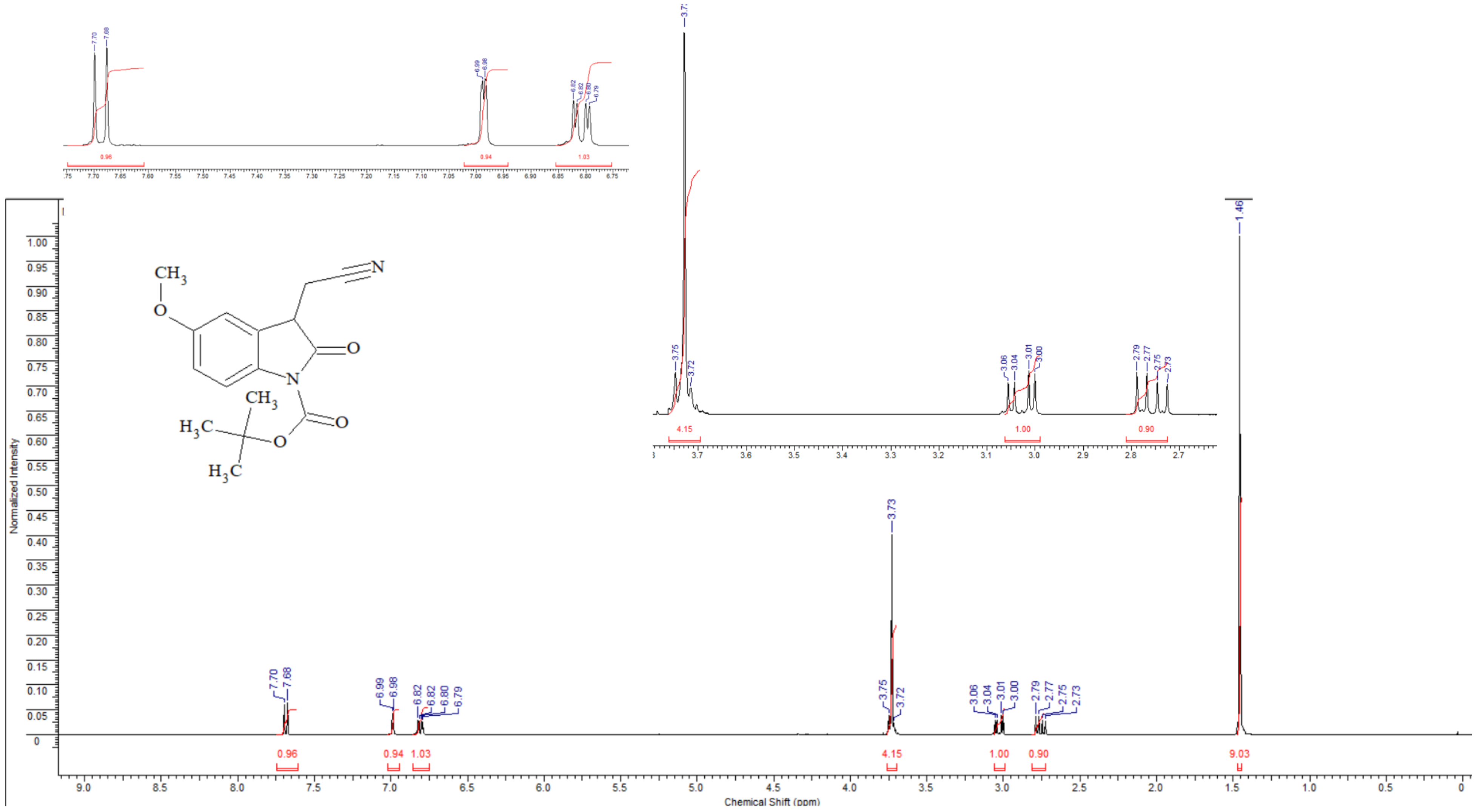

S13 13C NMR spectrum of Tert-butyl 5-methoxy-3-(cyanomethyl)-2-oxo-2,3-dihydro-1H-indol-1-carboxylate (**4l**)

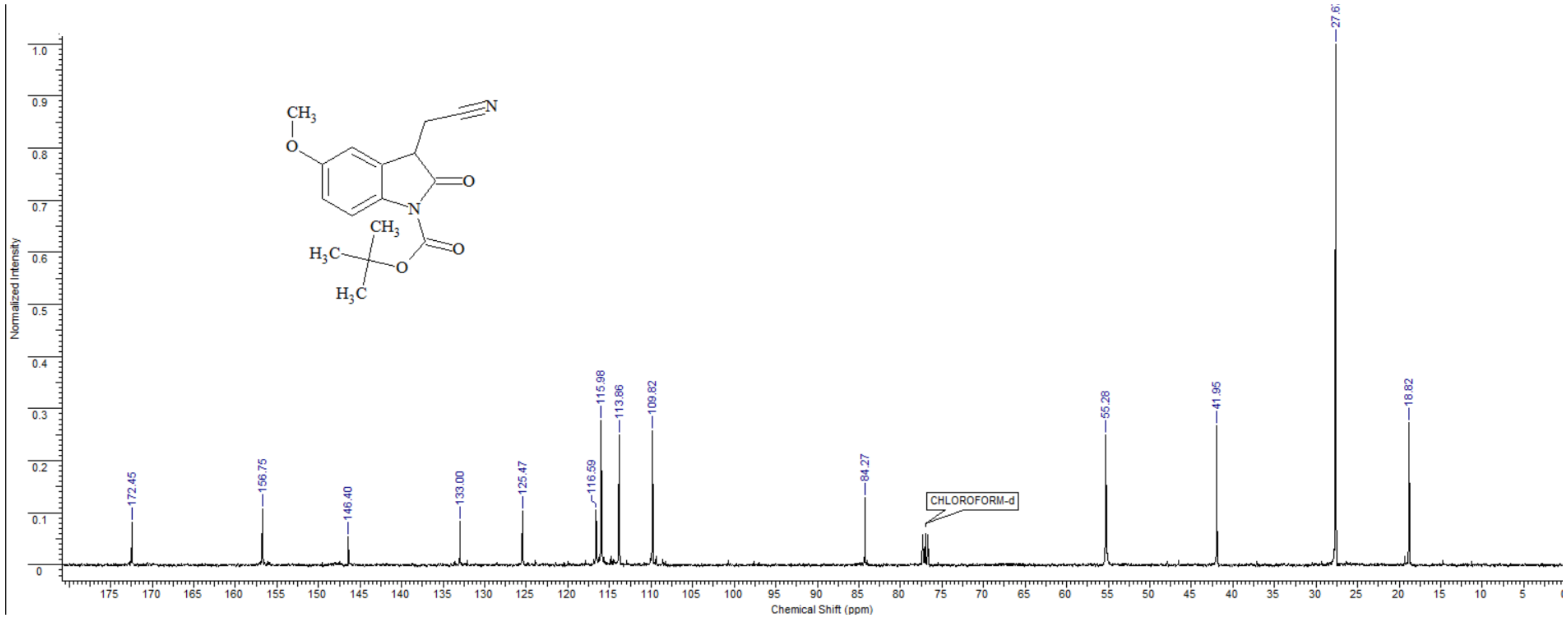

S14 1H NMR spectrum of 1,3-Di-tert-butyl 3-cyanomethyl-2-oxo-2,3-dihydro-1H-indol-1,3-dicarboxylate (**4m**)

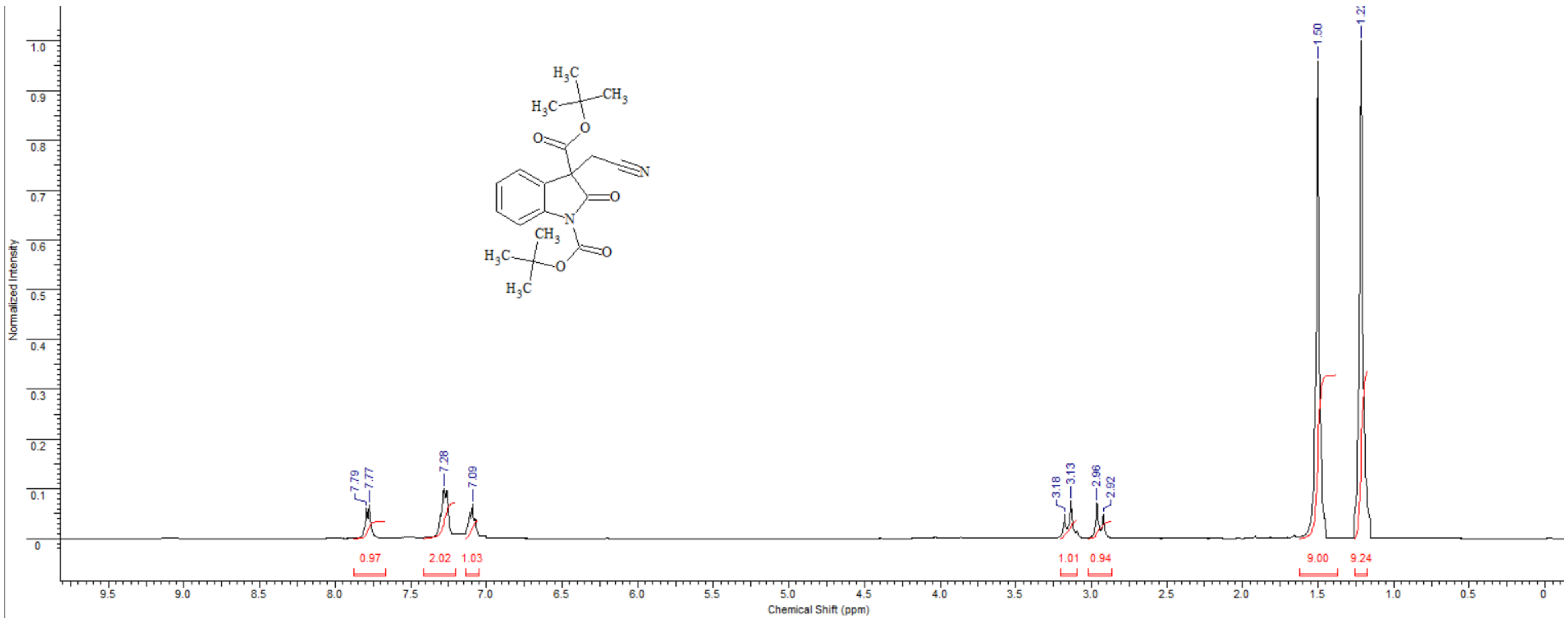

S15. 13C NMR spectrum of 1,3-Di-tert-butyl 3-cyanomethyl-2-oxo-2,3-dihydro-1H-indol-1,3-dicarboxylate (**4m**)

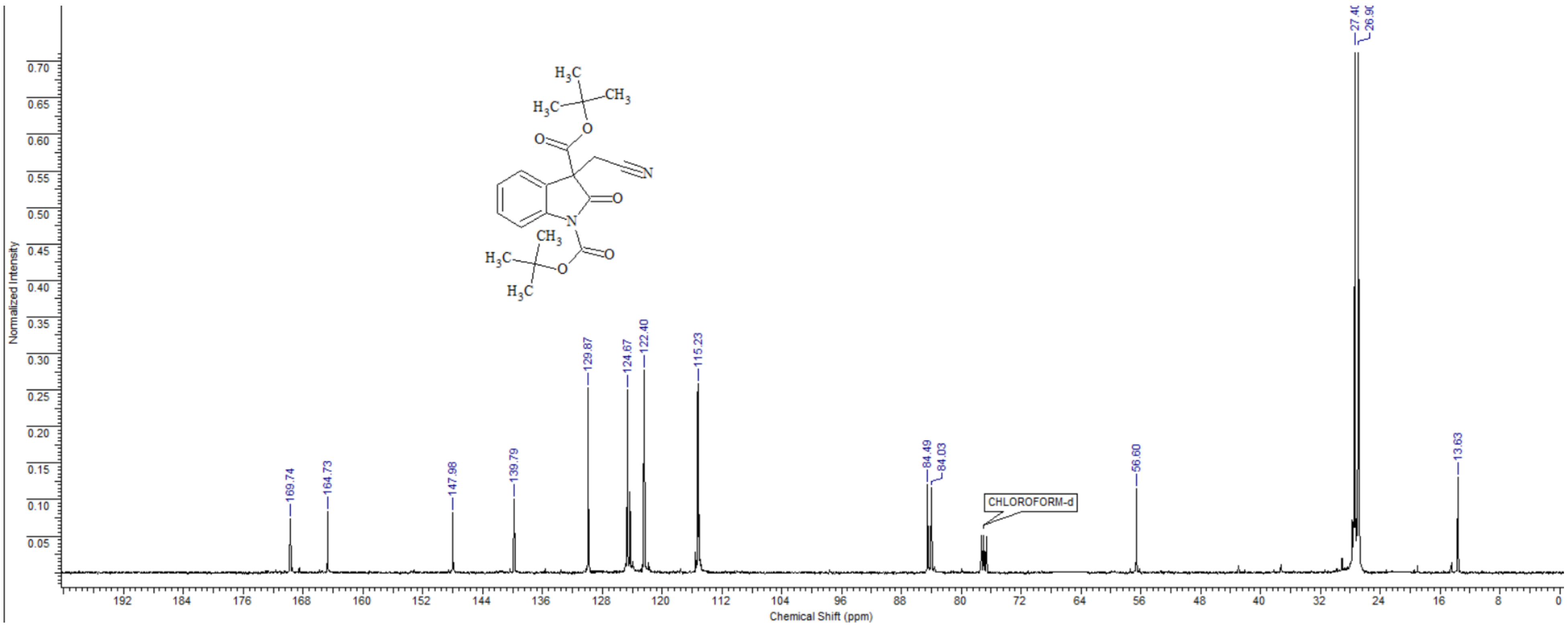

S16 1H NMR spectrum of 2-(1,3-Dimethyl-2-oxo-2,3-dihydro-1H-indol-3-yl)acetonitrile (**5a**)

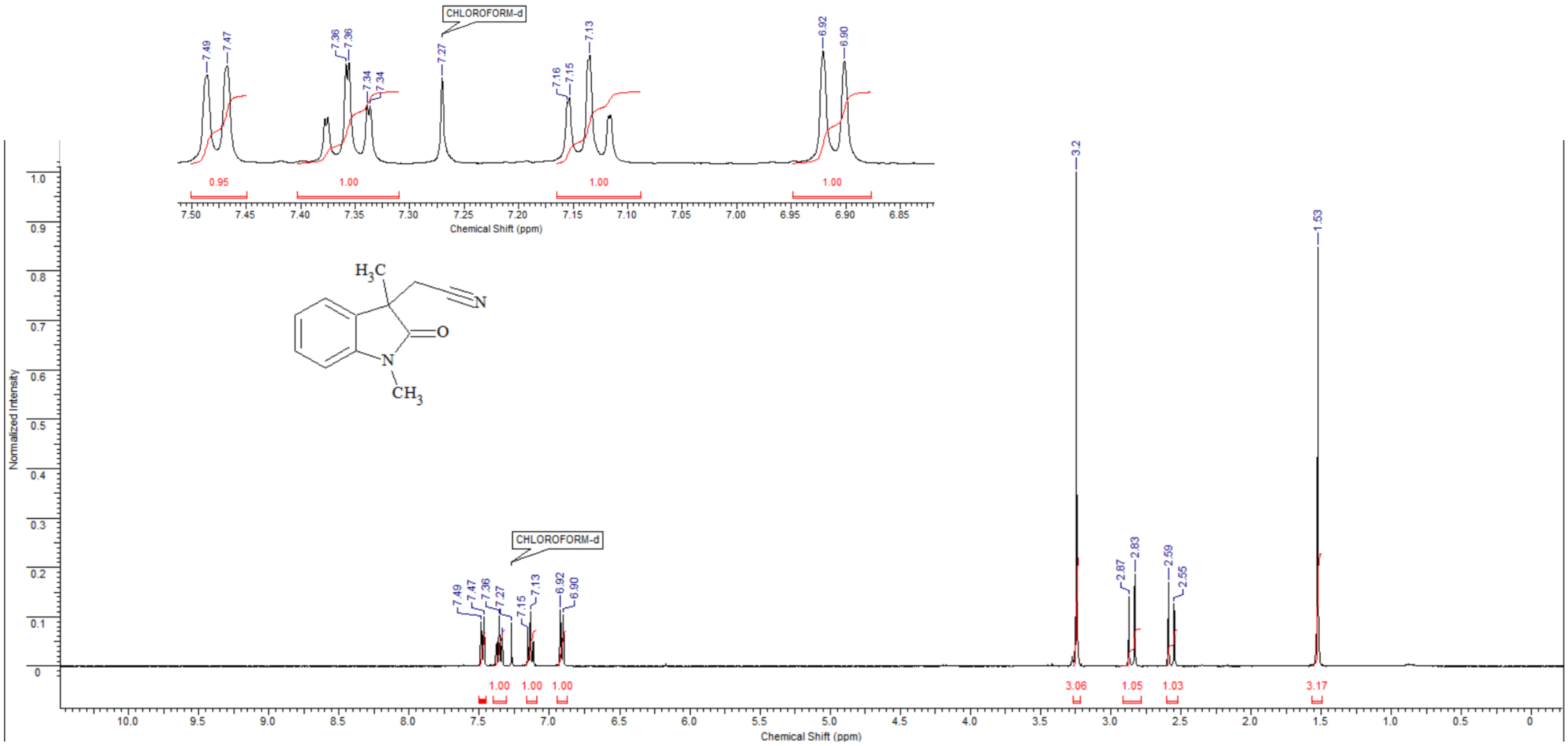

S17 13C NMR spectrum of 2-(1,3-Dimethyl-2-oxo-2,3-dihydro-1H-indol-3-yl)acetonitrile (**5a**)

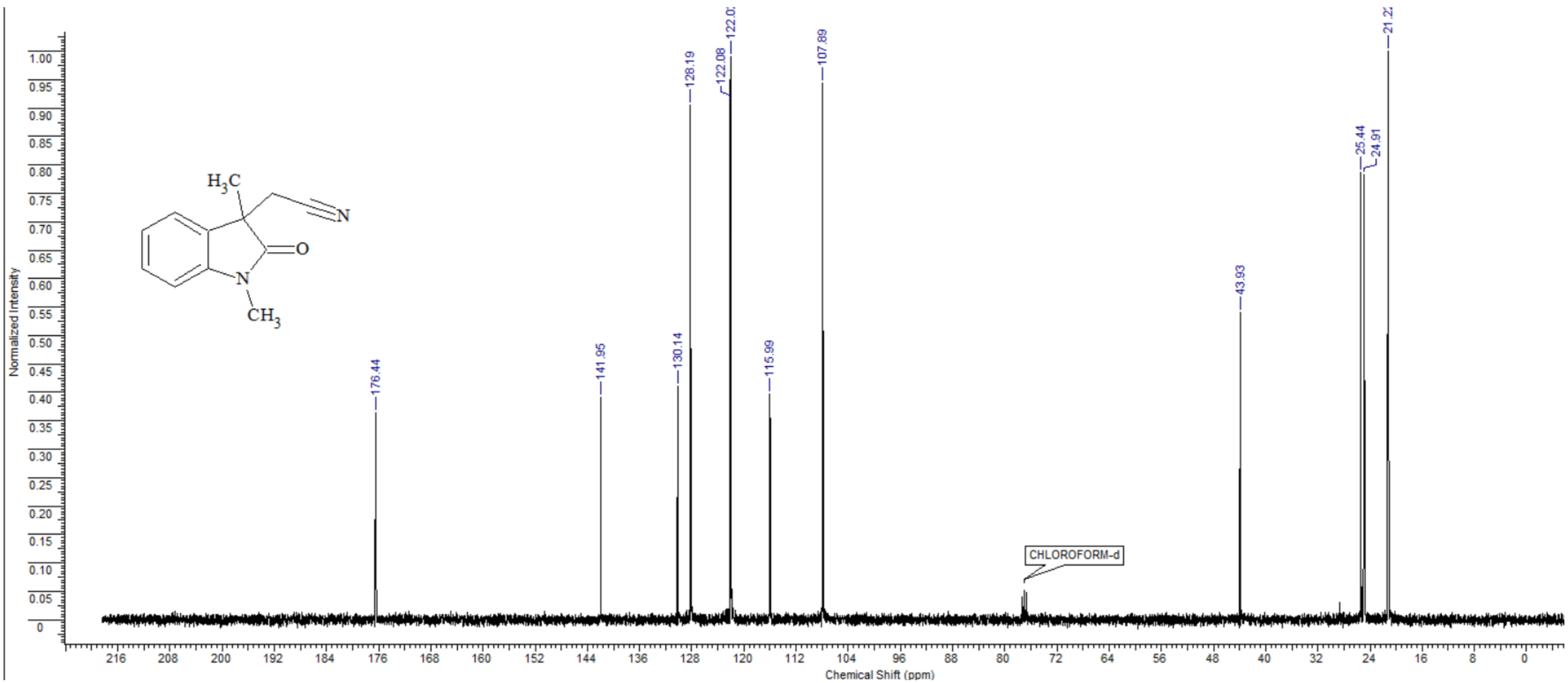

S18 1H NMR spectrum of 2-(1-Benzyl-3-methyl-2-oxo-2,3-dihydro-*1H*-indol-3-yl)acetonitrile (**5b**)

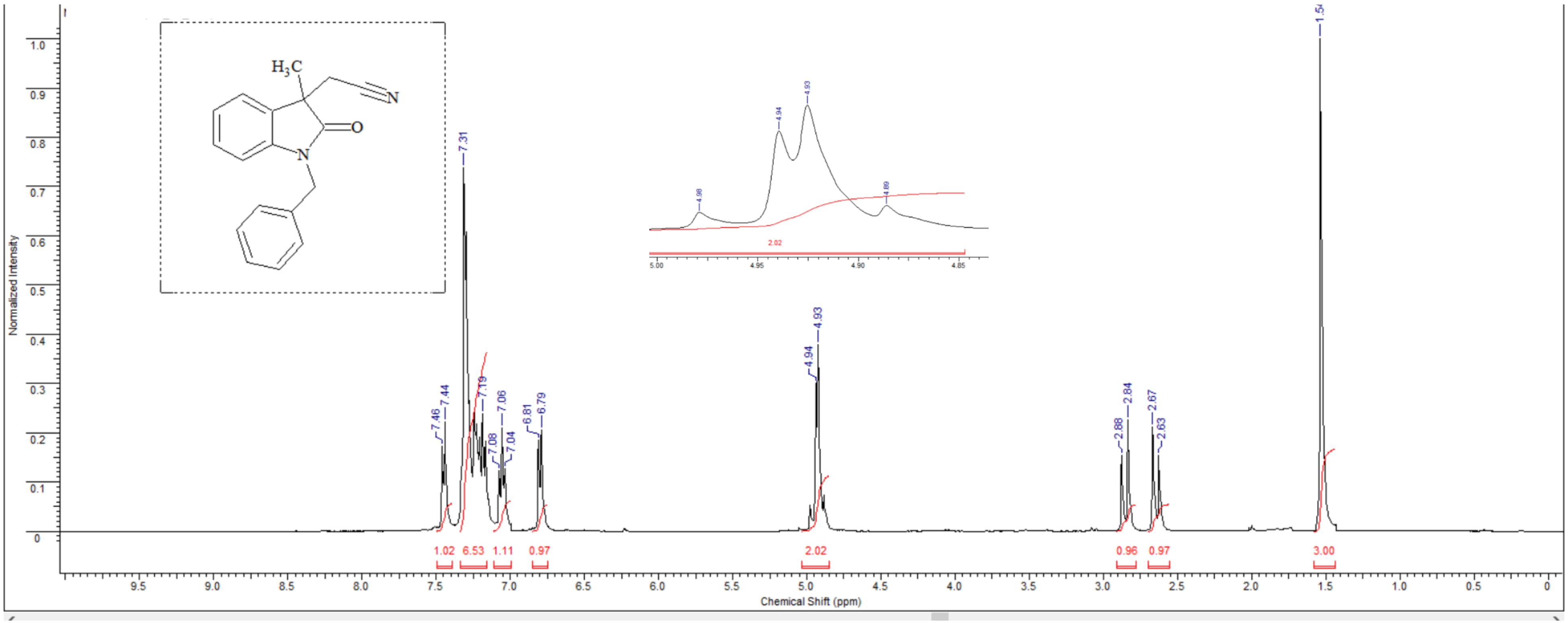

S19 13C NMR spectrum of 2-(1-Benzyl-3-methyl-2-oxo-2,3-dihydro-*1H*-indol-3-yl)acetonitrile (**5b**)

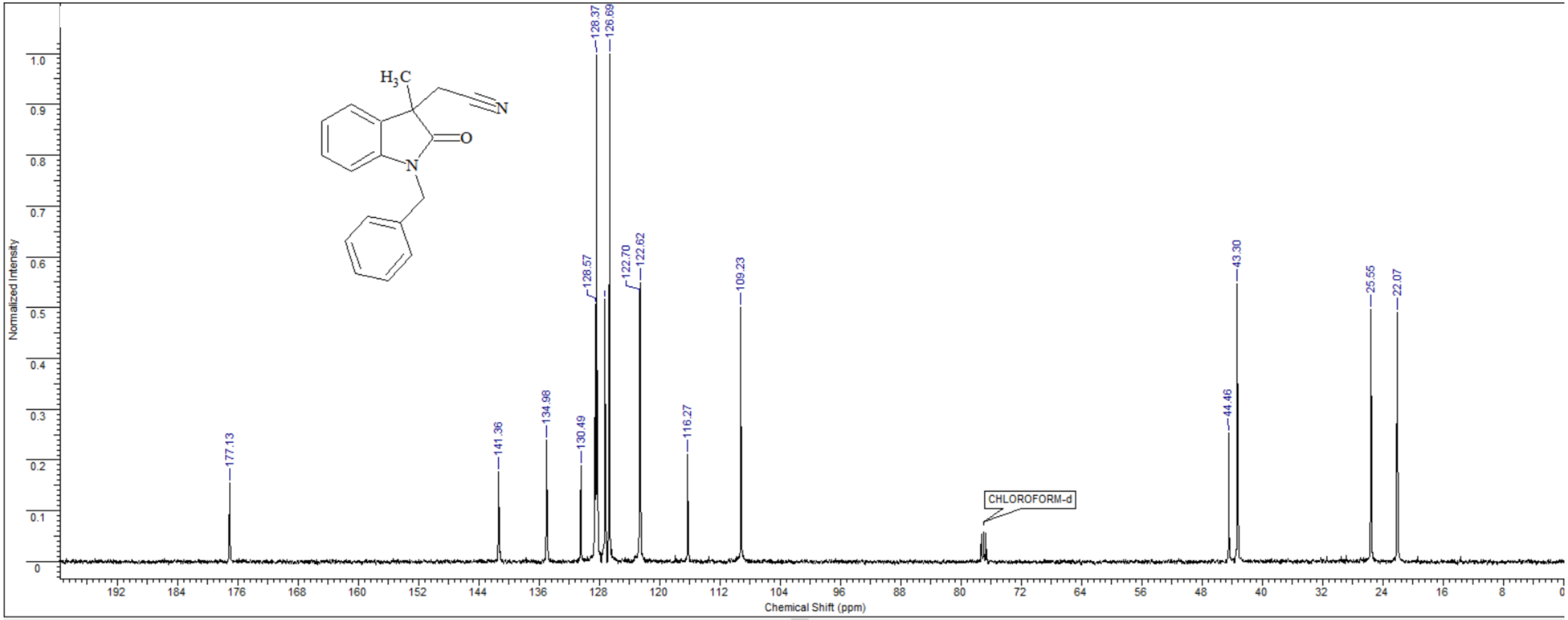

S20 1H NMR spectrum of 2-(1-Methyl-3-cyanomethyl-2-oxo-2,3-dihydro-1H-indol-3-yl)acetonitrile (**5d**)

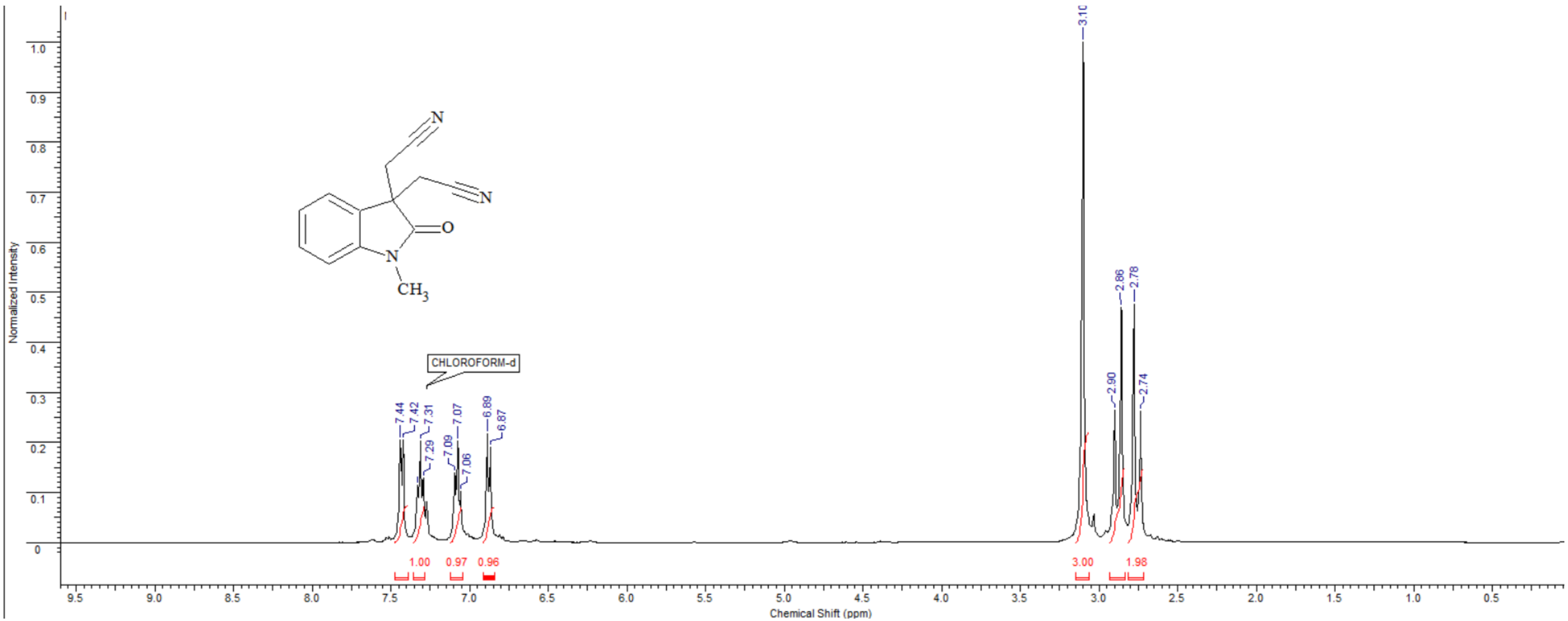

S21 13C NMR spectrum of 2-(1-Methyl-3-cyanomethyl-2-oxo-2,3-dihydro-1H-indol-3-yl)acetonitrile (**5d**)

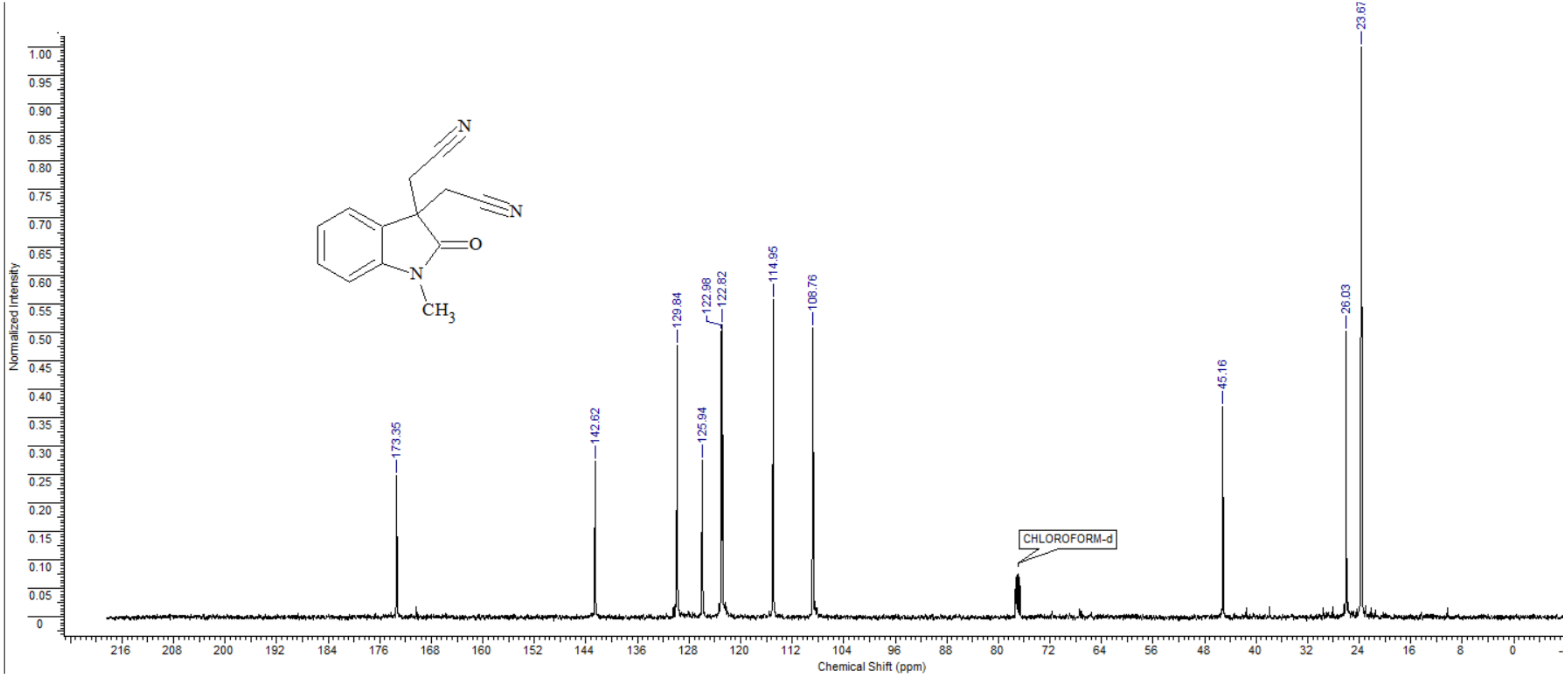

S22 1H NMR spectrum of 2-(1,3-Dimethyl-5-methoxy-2-oxo-2,3-dihydro-1H-indol-3-yl)acetonitrile (**5e**)

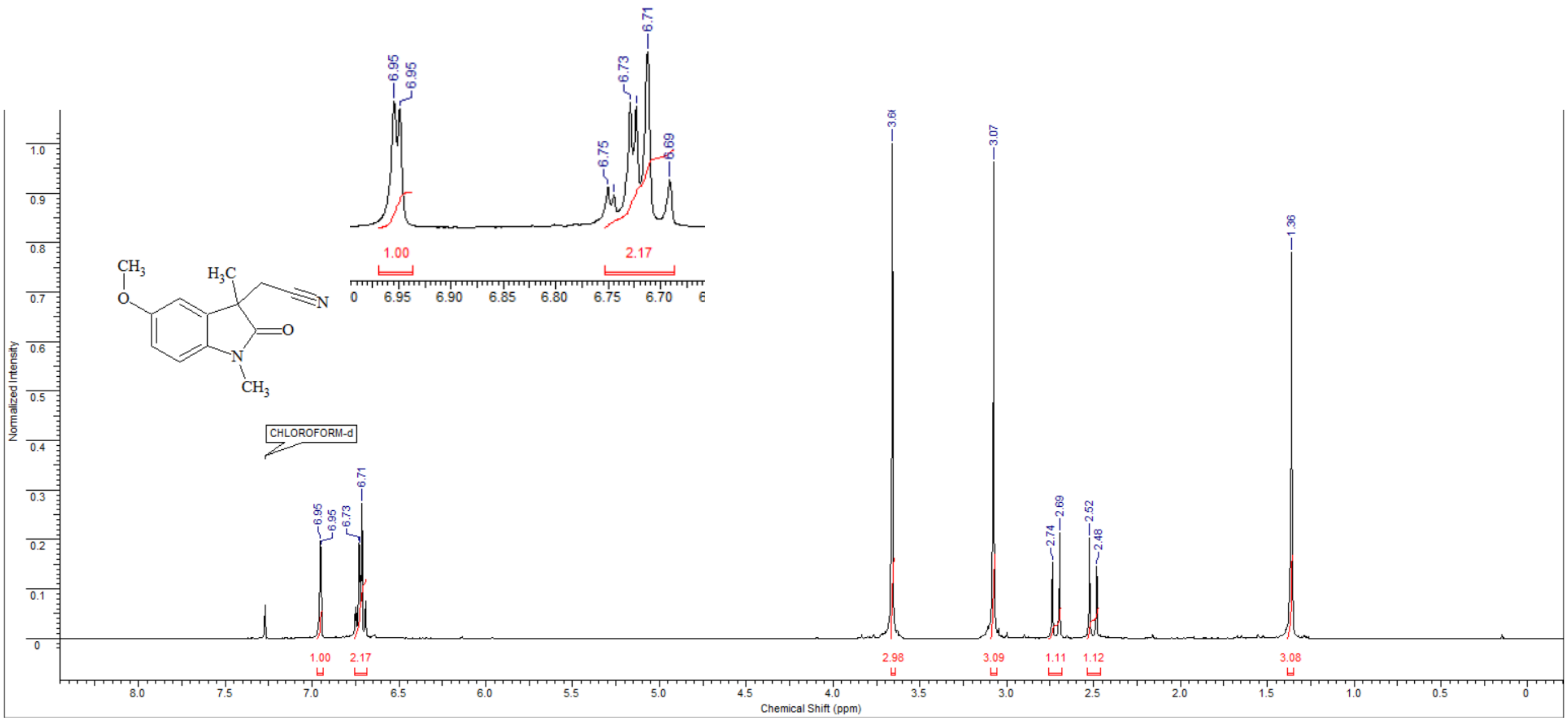

S23 13C NMR spectrum of 2-(1,3-Dimethyl-5-methoxy-2-oxo-2,3-dihydro-1H-indol-3-yl)acetonitrile (**5e**)

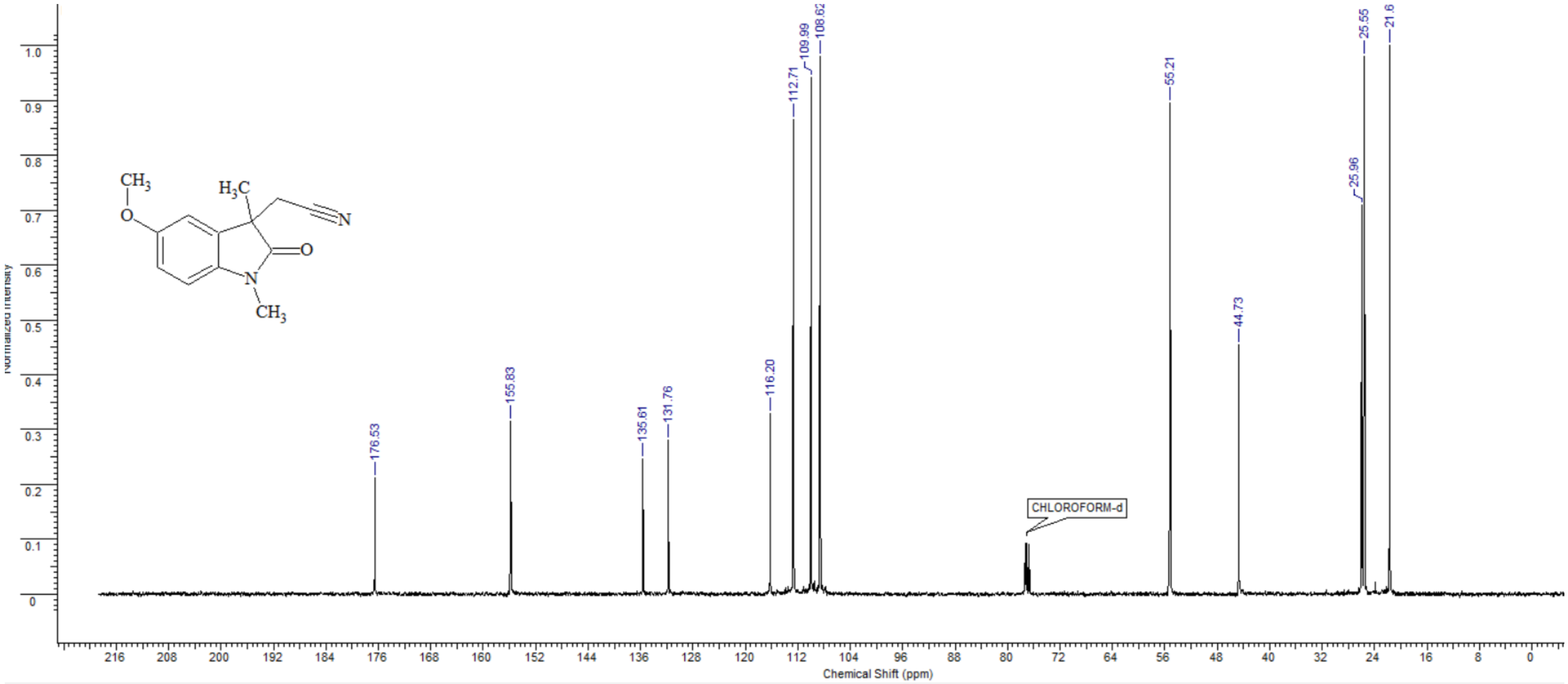

S24 1H NMR spectrum of 2-(1-Benzyl-3-methyl-5-methoxy-2-oxo-2,3-dihydro-*1H*-indol-3-yl)acetonitrile (**5f**)

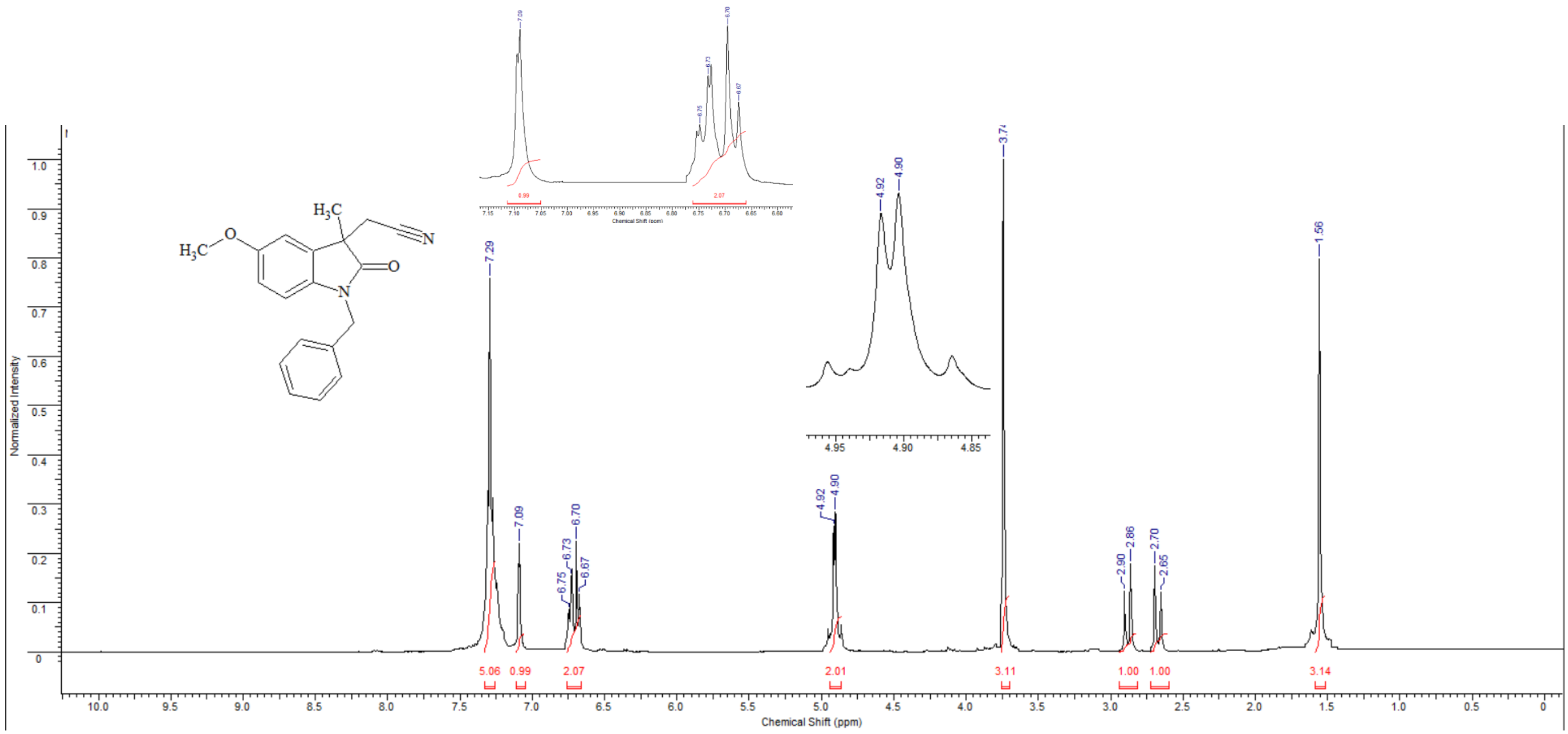

S25 13C NMR spectrum of 2-(1-Benzyl-3-methyl-5-methoxy-2-oxo-2,3-dihydro-*1H*-indol-3-yl)acetonitrile (**5f**)

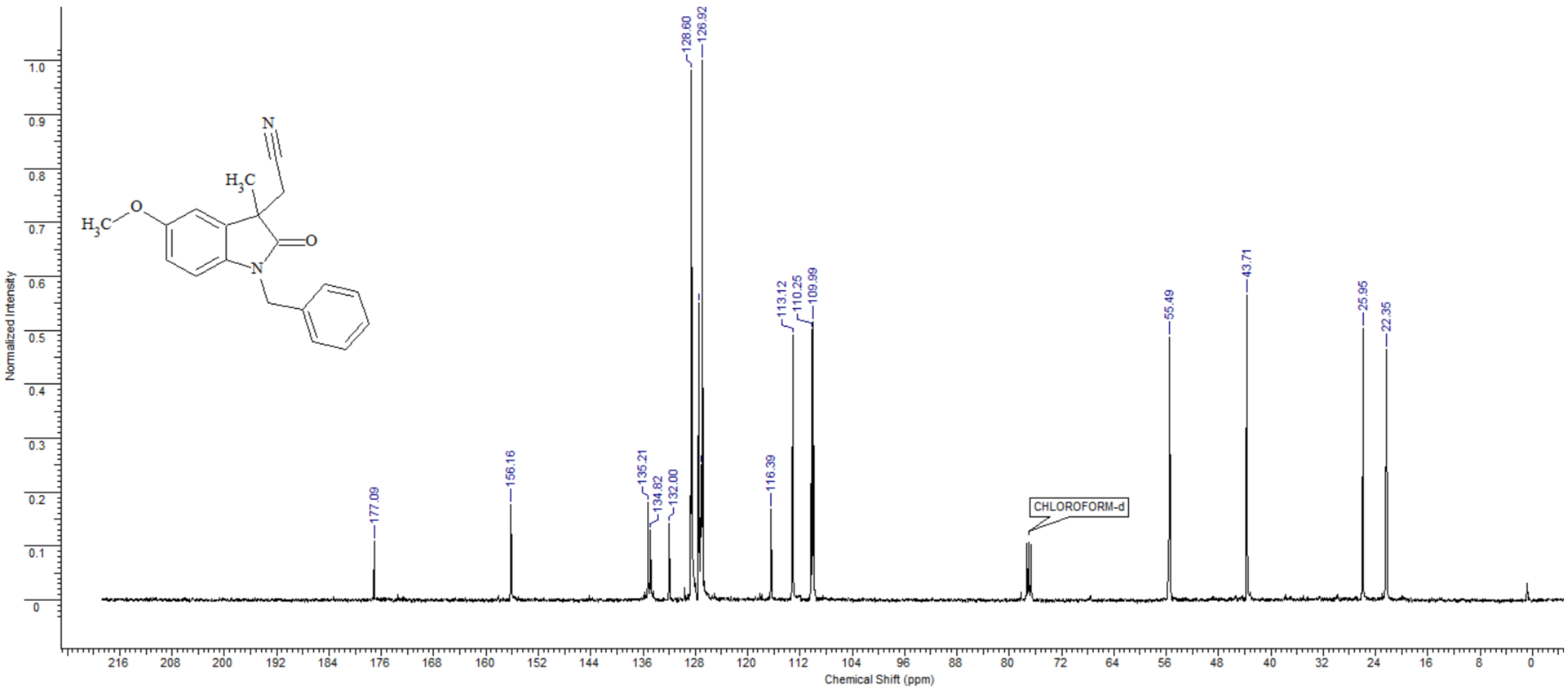

S26 1H NMR spectrum of 2-(3-Ethyl-1-methyl-5-methoxy-2-oxo-2,3-dihydro-1H-indol-3-yl)acetonitrile (**5g**)

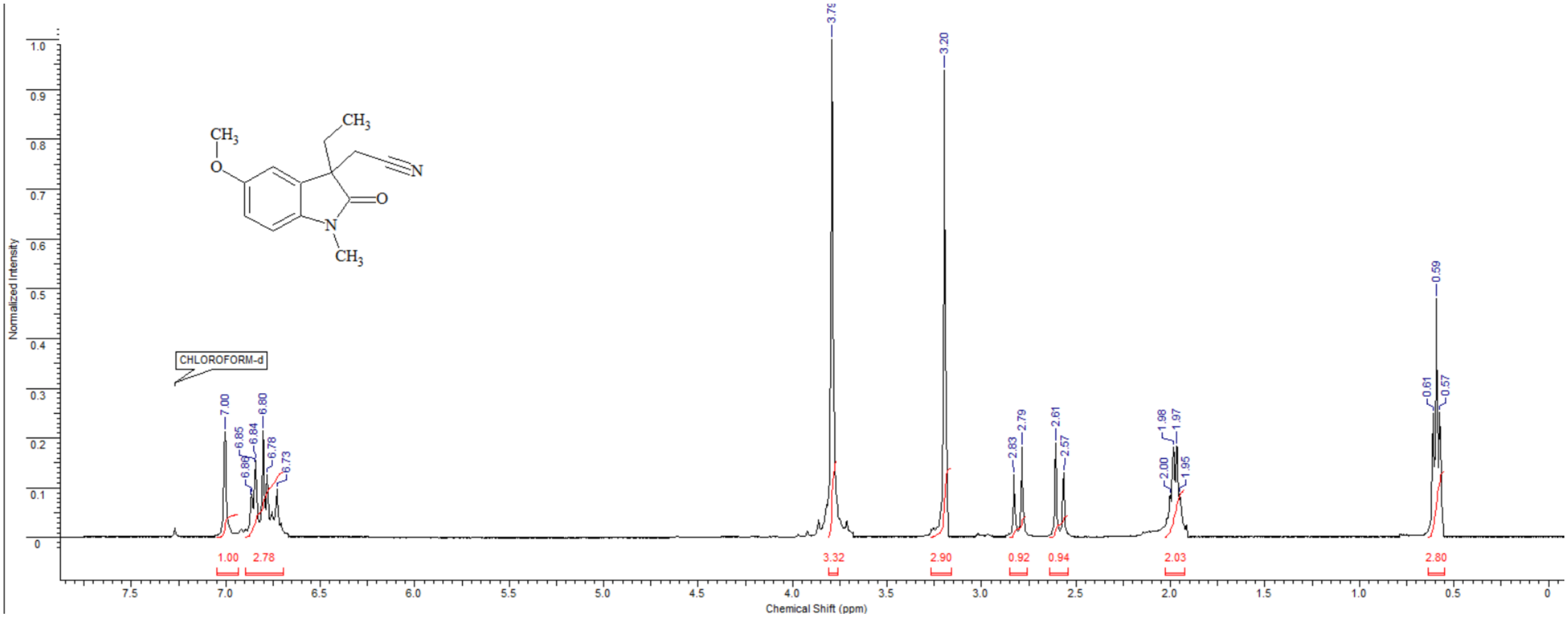

S27 13C NMR spectrum of 2-(3-Ethyl-1-methyl-5-methoxy-2-oxo-2,3-dihydro-1H-indol-3-yl)acetonitrile (**5g**)

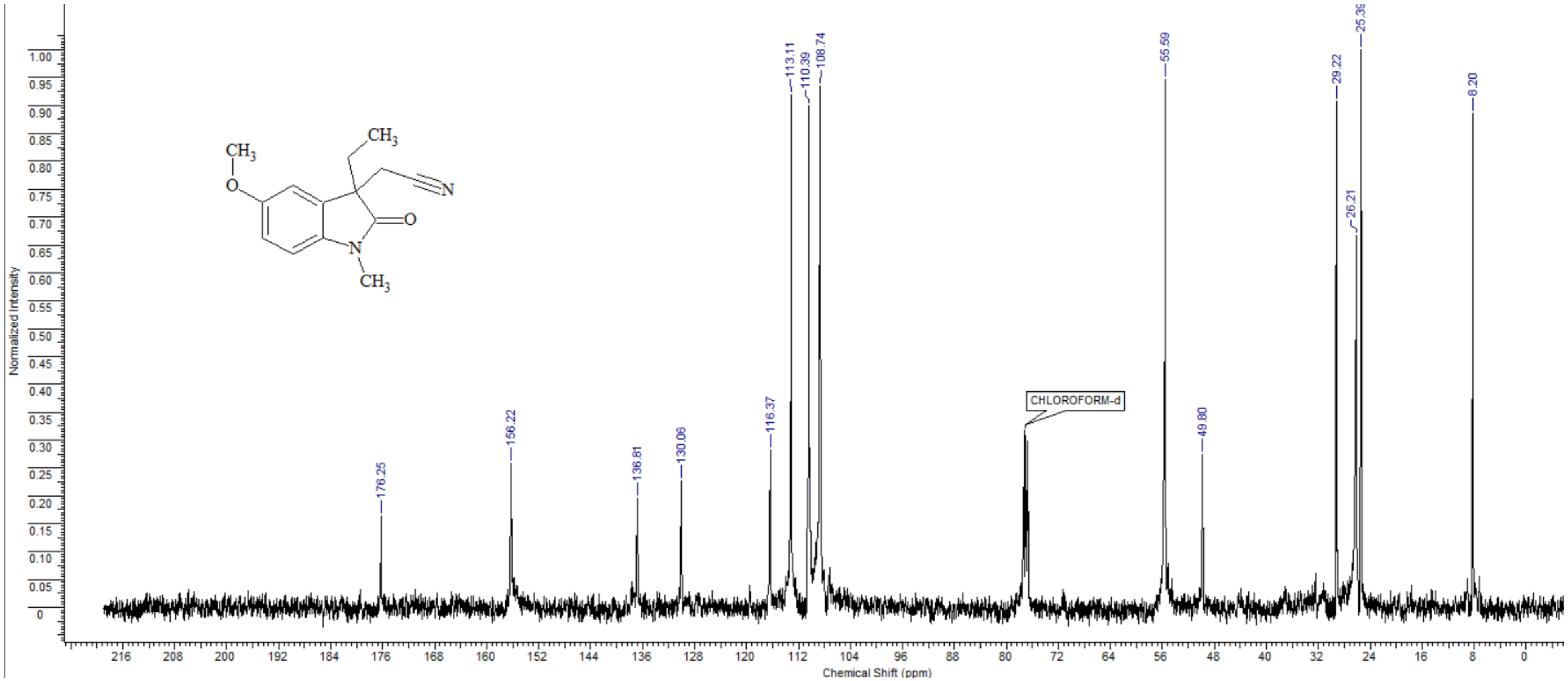

S28 1H NMR spectrum of 2-(1-Methyl-5-methoxy-3-(cyanomethyl)-2-oxo-2,3-dihydro-1H-indol-3-yl)acetonitrile (**5h**)

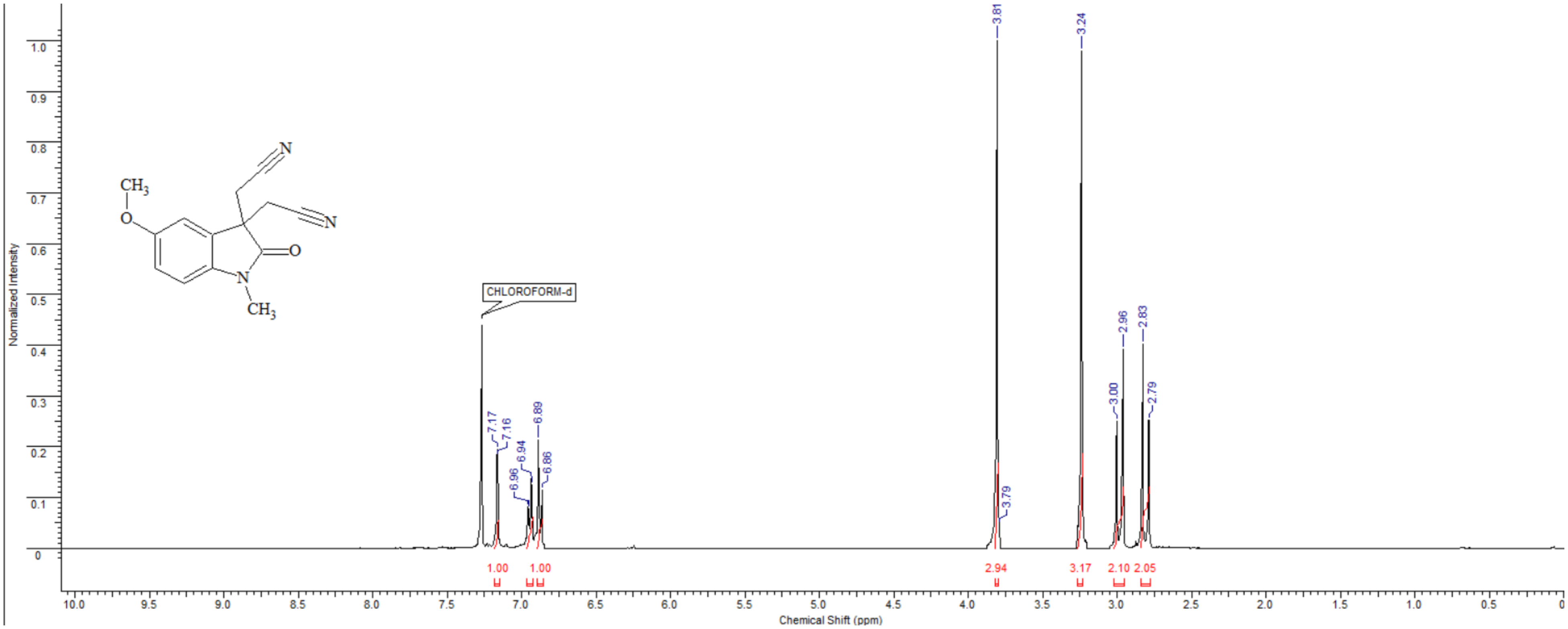

S29 13C NMR spectrum of 2-(1-Methyl-5-methoxy-3-(cyanomethyl)-2-oxo-2,3-dihydro-1H-indol-3-yl)acetonitrile (**5h**)

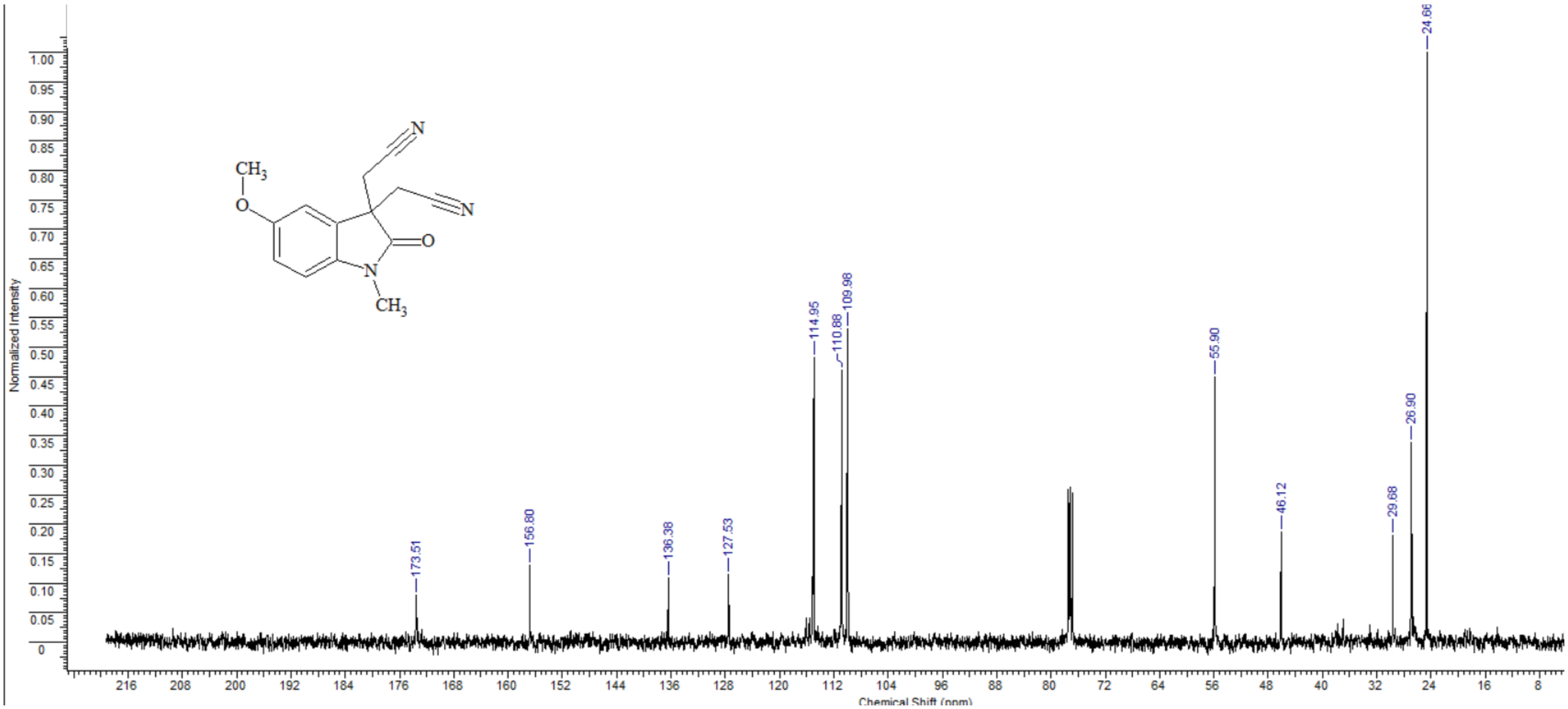

S30 1H NMR spectrum of 2-(5-Bromo-1,3-dimethyl-2-oxo-2,3-dihydro-1H-indol-3-yl)acetonitrile (**5j**)

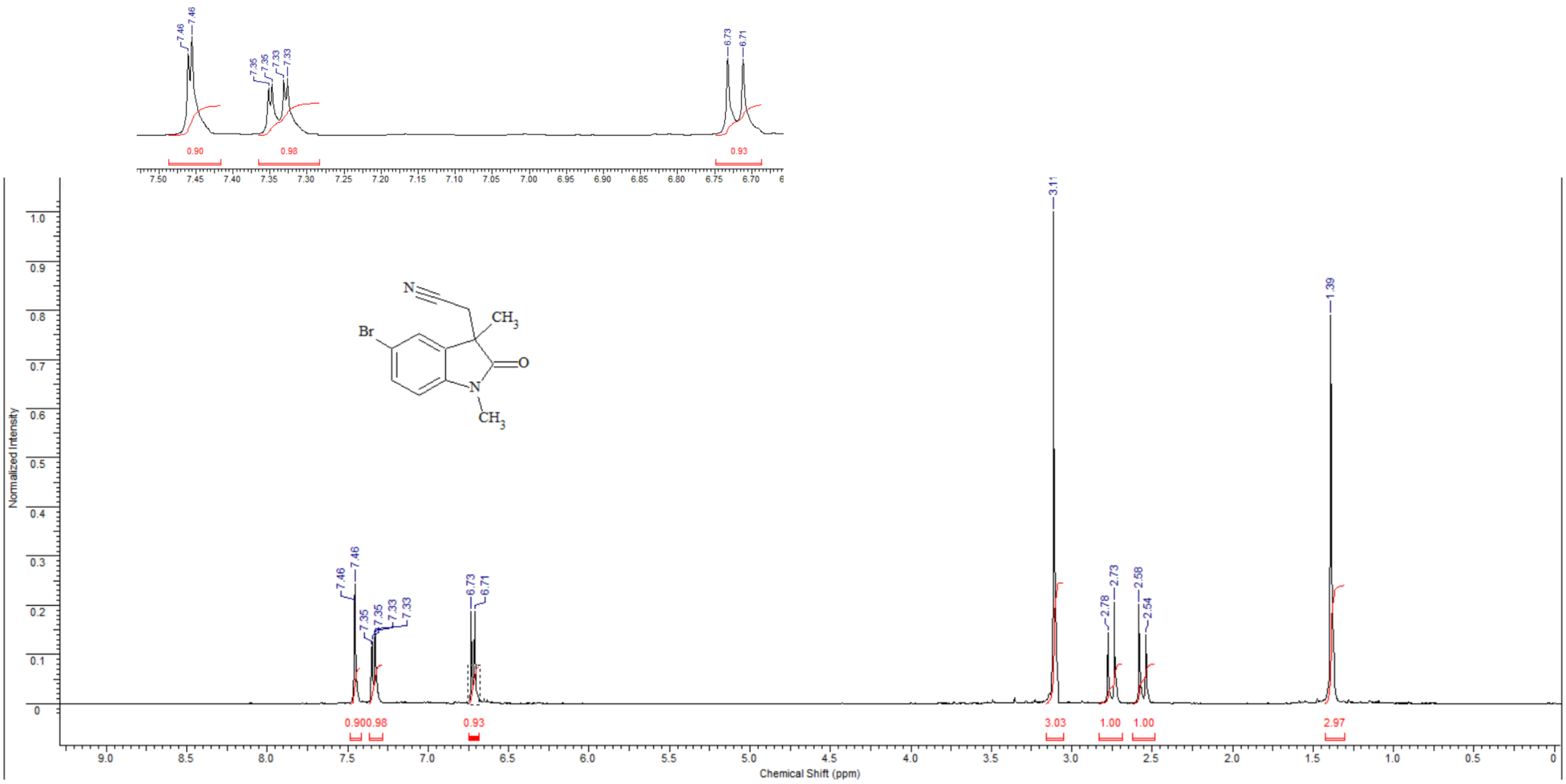

S31 13C NMR spectrum of 2-(5-Bromo-1,3-dimethyl-2-oxo-2,3-dihydro-1H-indol-3-yl)acetonitrile (**5j**)

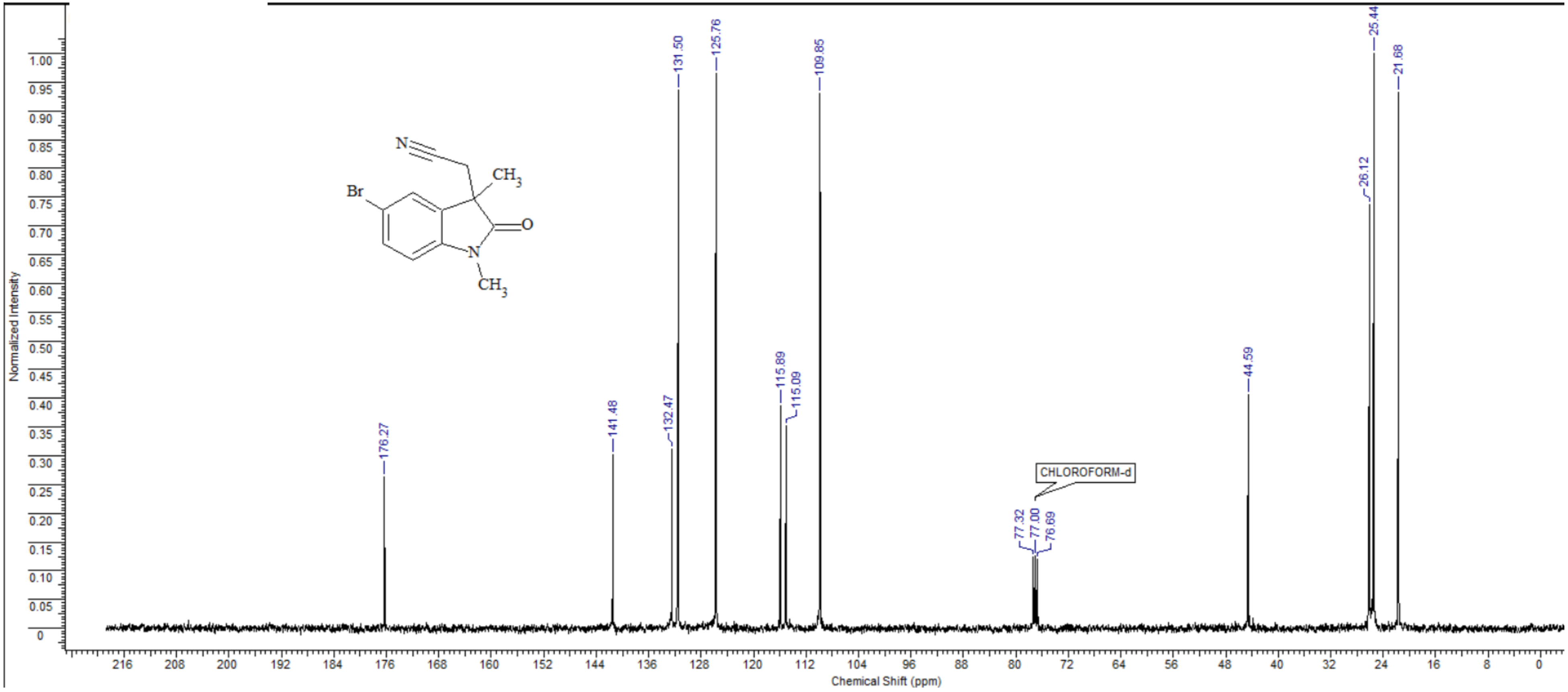

S32 1H NMR spectrum of 2-(1,3,6,7-Tetramethyl-2-oxo-2,3-dihydro-*1H*-indol-3-yl)acetonitrile (**5k**)

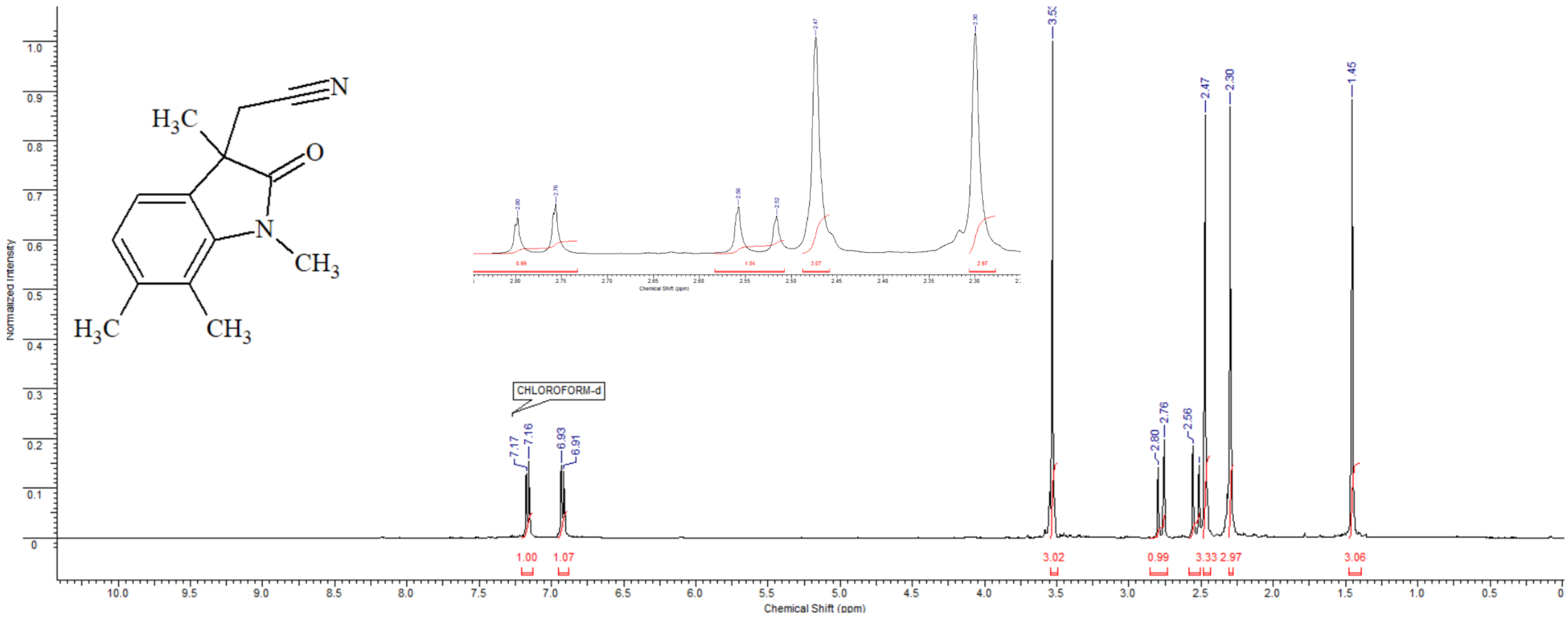

S33 1H NMR spectrum of Tert-butyl 3-(cyanomethyl)-3-methyl-2-oxo-2,3-dihydro-*1H*-indole-1-carboxylate (**5l**)

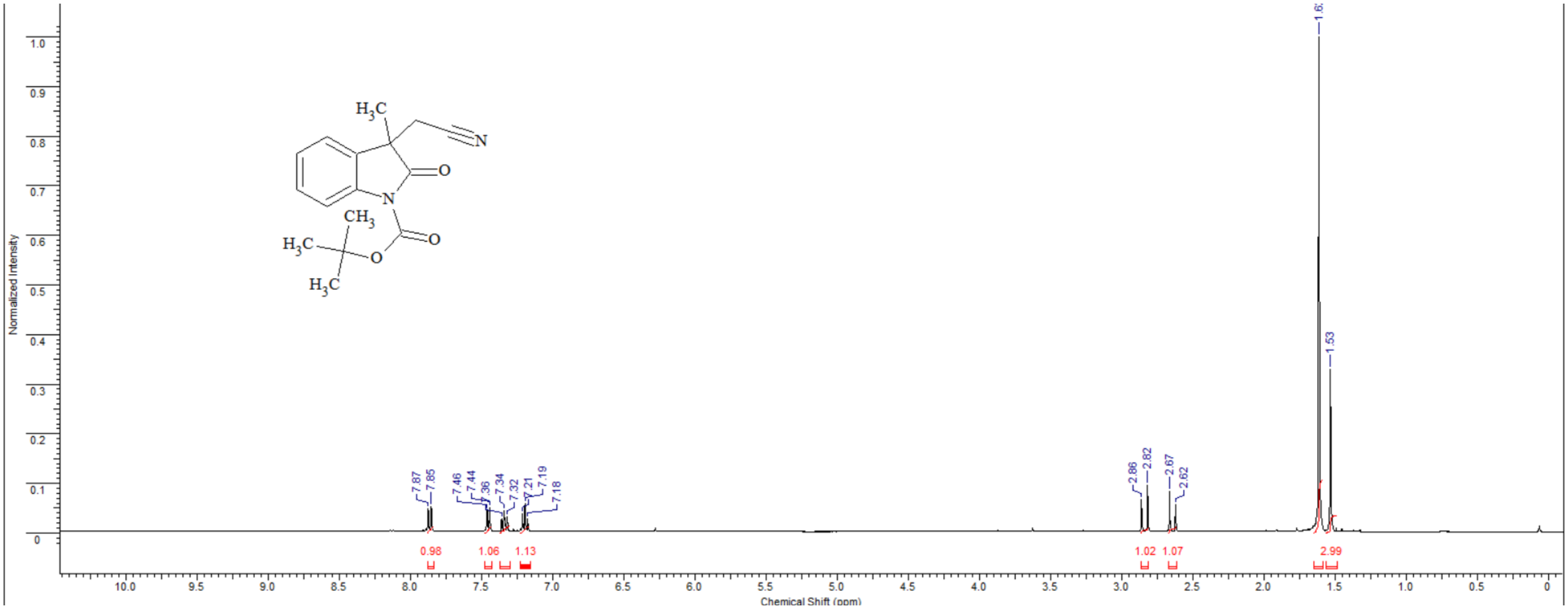

S34 13C NMR spectrum of Tert-butyl 3-(cyanomethyl)-3-methyl-2-oxo-2,3-dihydro-*1H*-indole-1-carboxylate (**5l**)

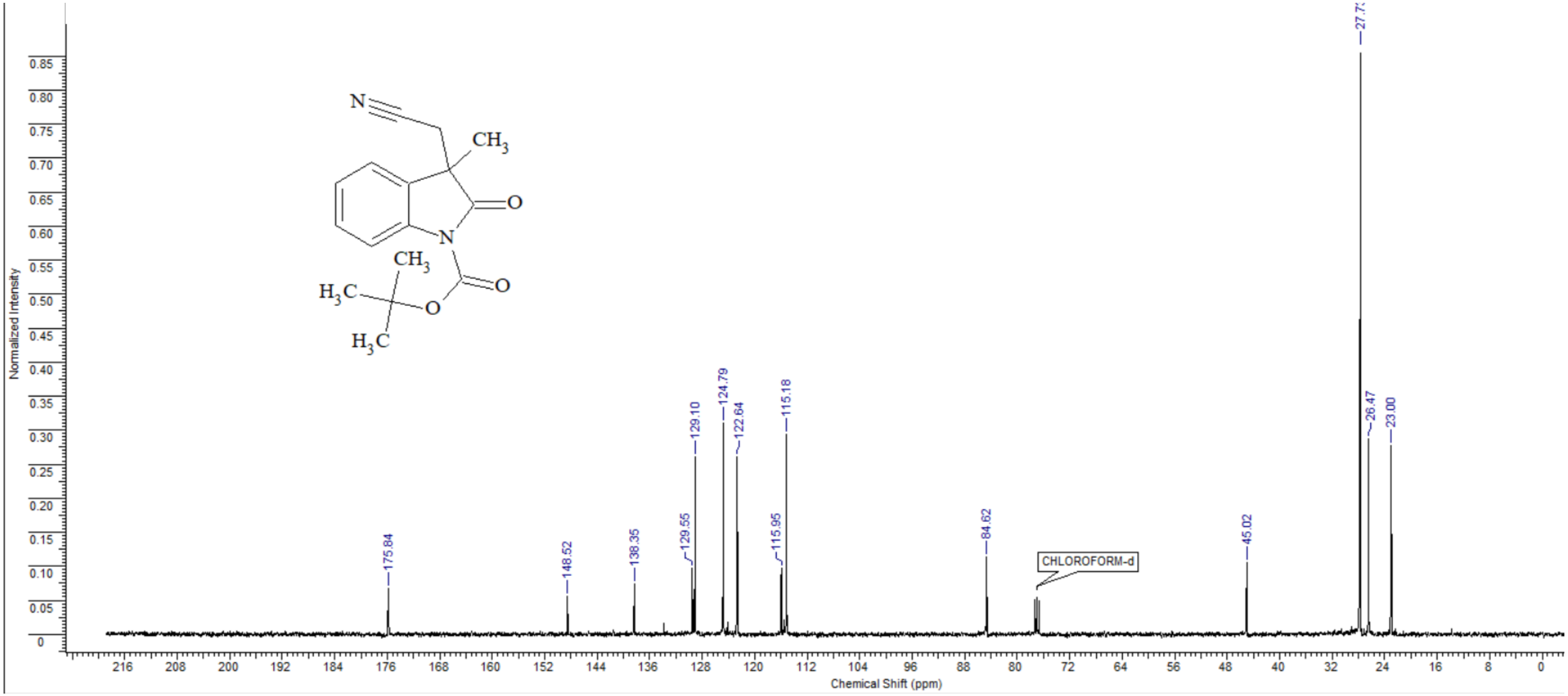

S35 1H NMR spectrum of (1R*,2R*)-6′,7′-Dimethyl-2'-oxo-1',2'-dihydrospiro[cyclopropane-1,3'-indole]-3-carbonitrile (**6d**)

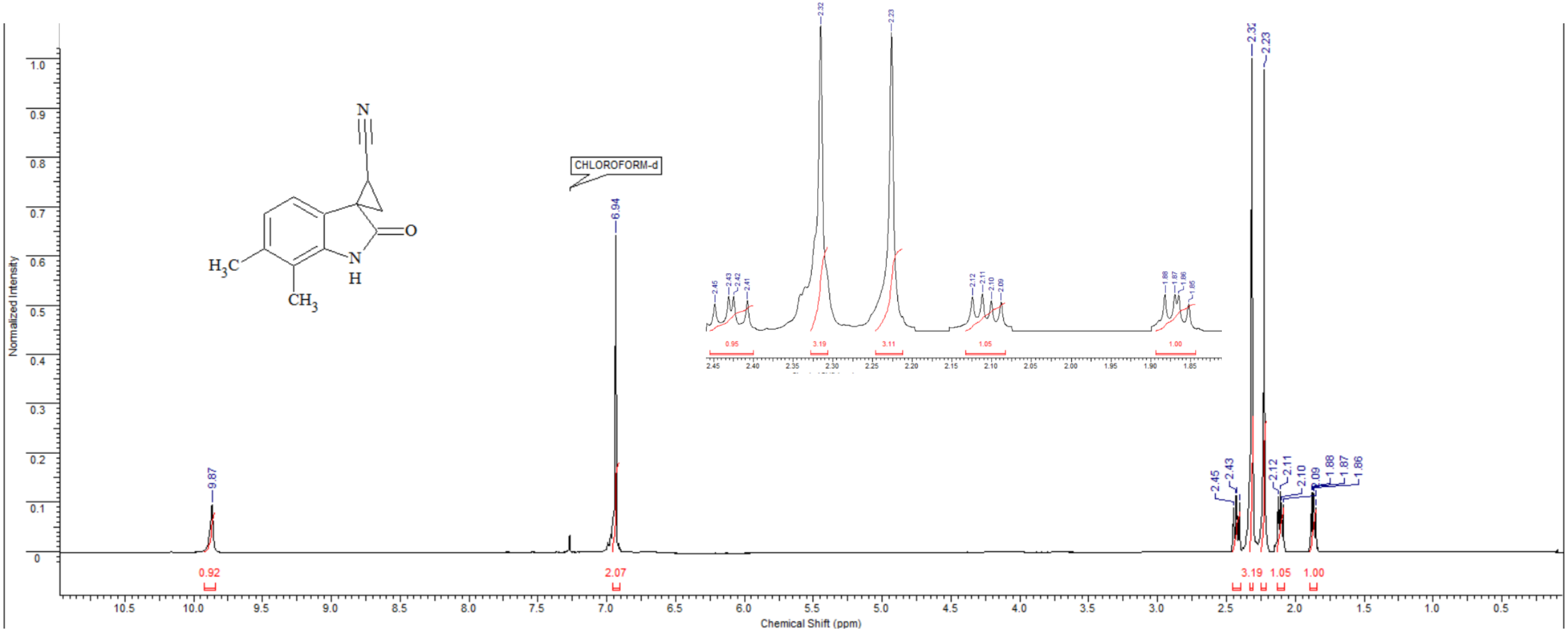

S36 13C NMR spectrum of (1R*,2R*)-6’,7’-Dimethyl-2'-oxo-1',2'-dihydrospiro[cyclopropane-1,3'-indole]-3-carbonitrile (**6d**)

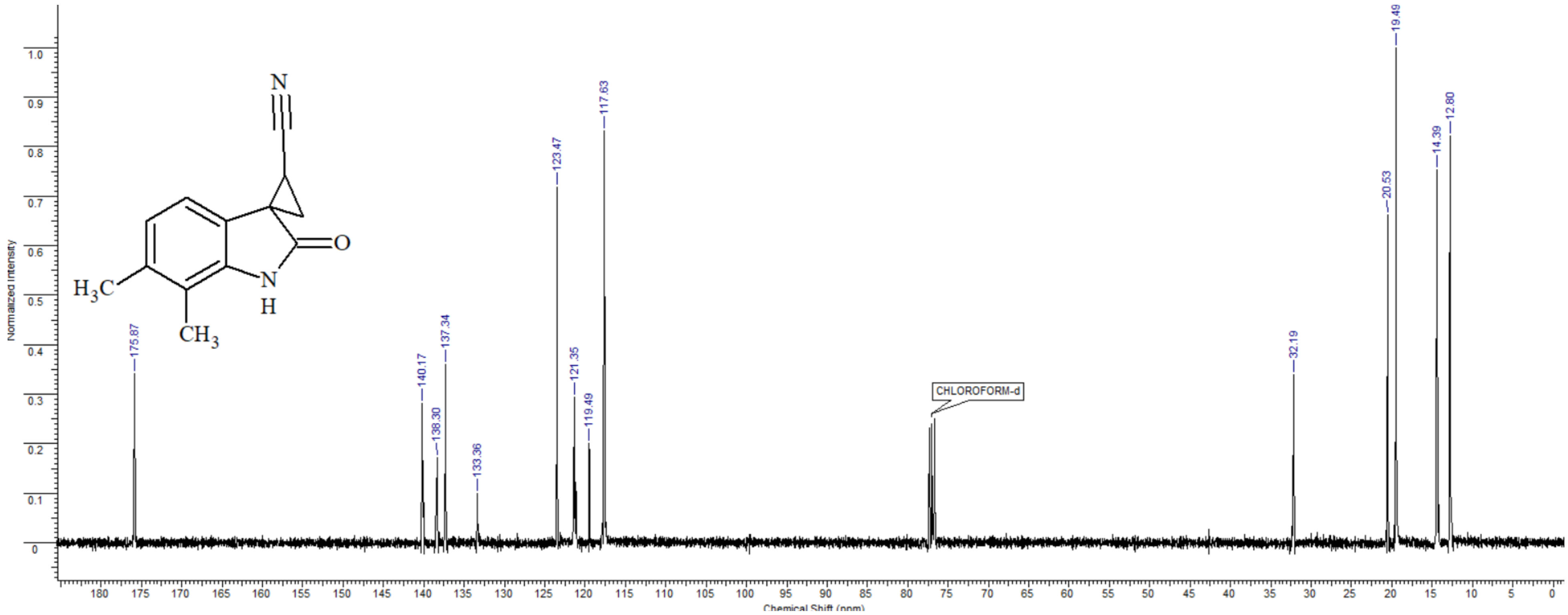

S37 1H NMR spectrum of 1’-Methyl-5’-methoxy-2’-oxo-1’,2’-dihydrospiro[cyclopropane-1,3’-indole]-3-carbonitrile (**6g**) (mixture of isomers)

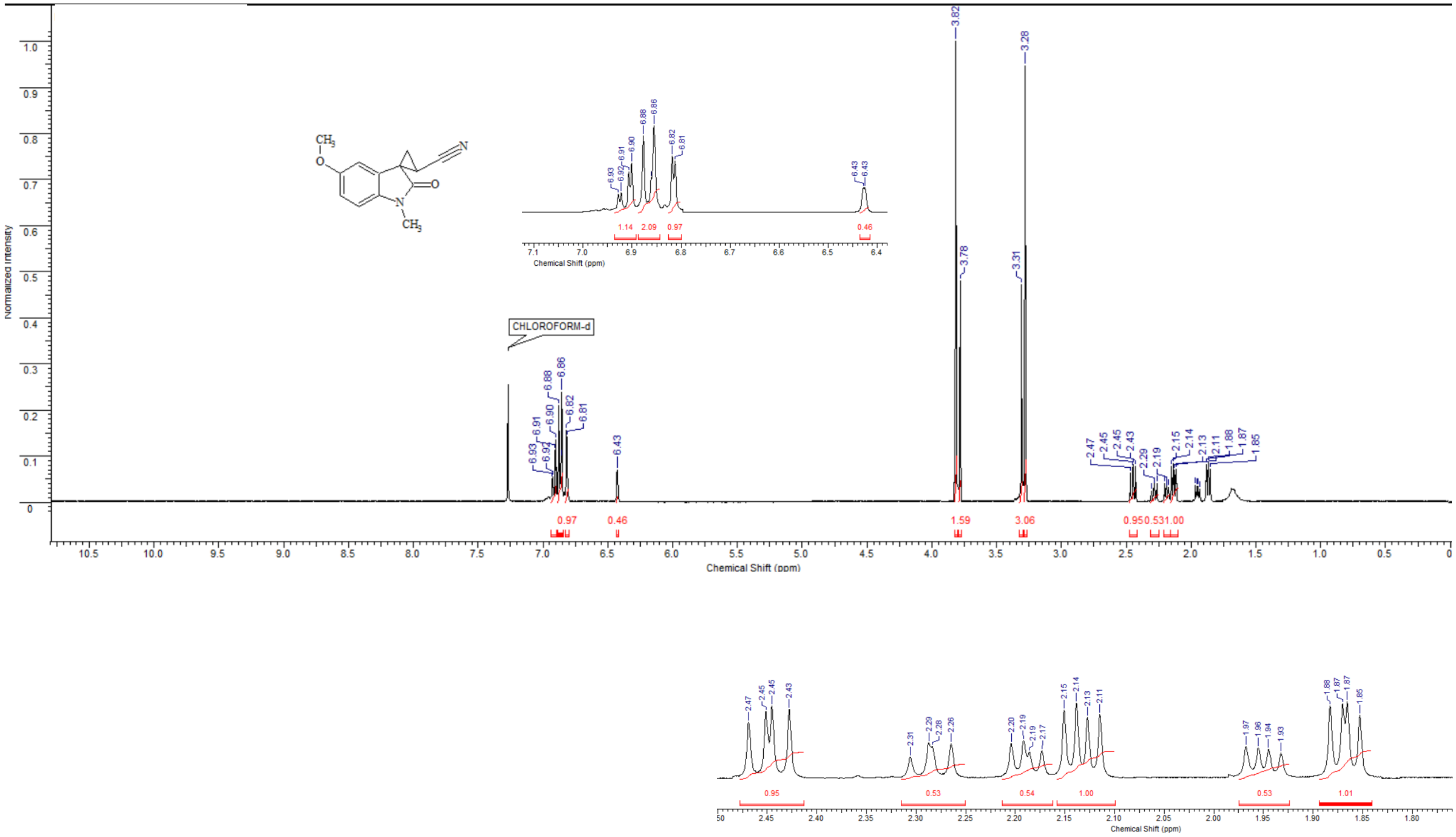

S38 13C NMR spectrum of 1'-Methyl-5'-methoxy-2'-oxo-1',2'-dihydrospiro[cyclopropane-1,3'-indole]-3-carbonitrile (**6g**) (single isomers)

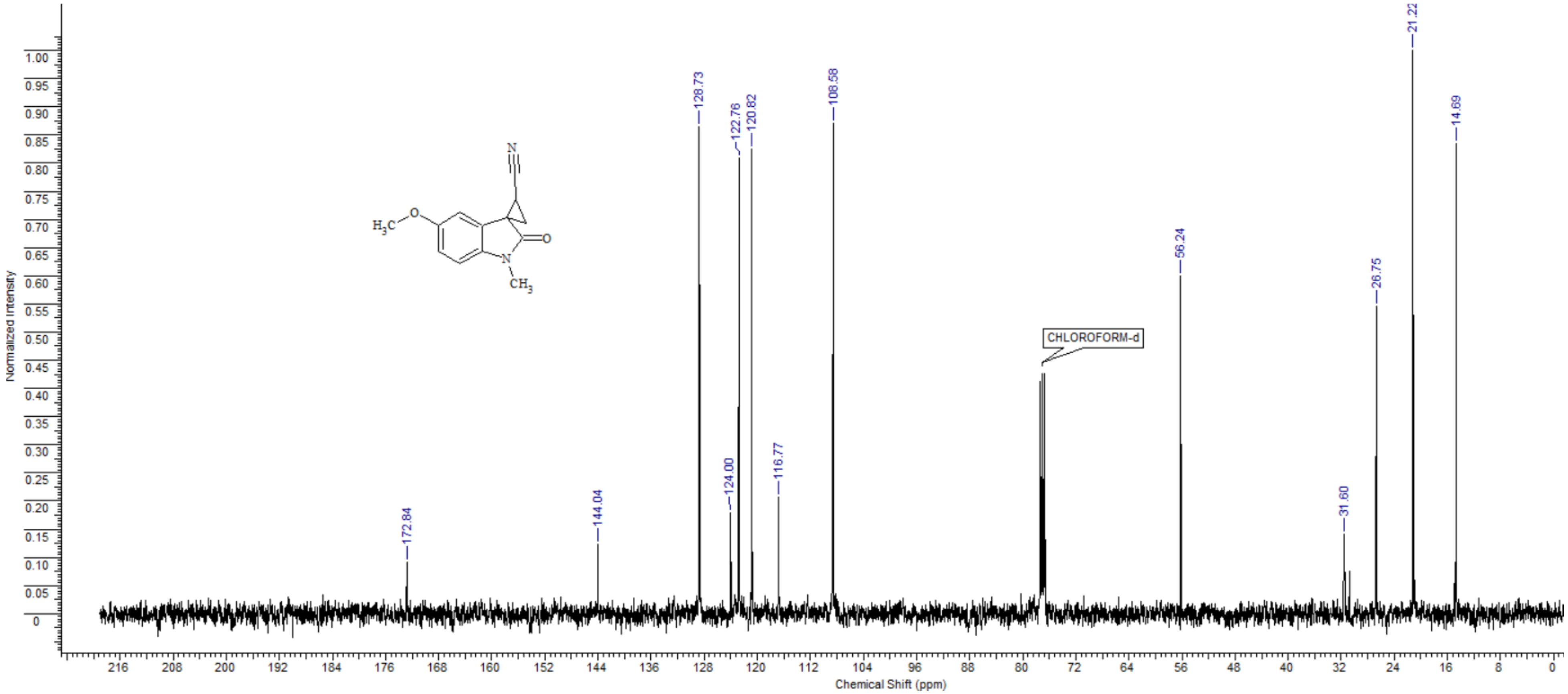

S39 1H NMR spectrum of 5'-Bromo-1-methyl-2'-oxo-1',2'-dihydrospiro[cyclopropane-1,3'-indole]-3-carbonitrile (**6i**)

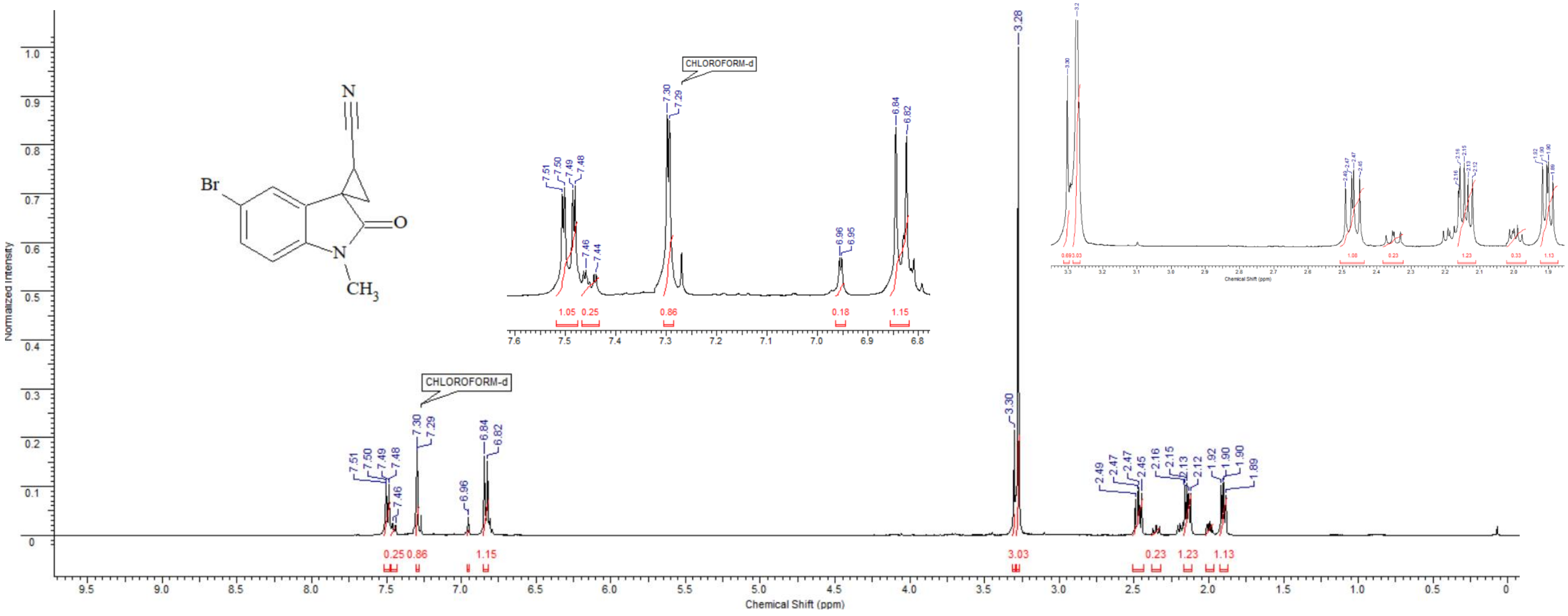

S40 13C NMR spectrum of 5'-Bromo-1-methyl-2'-oxo-1',2'-dihydrospiro[cyclopropane-1,3'-indole]-3-carbonitrile (**6i**)

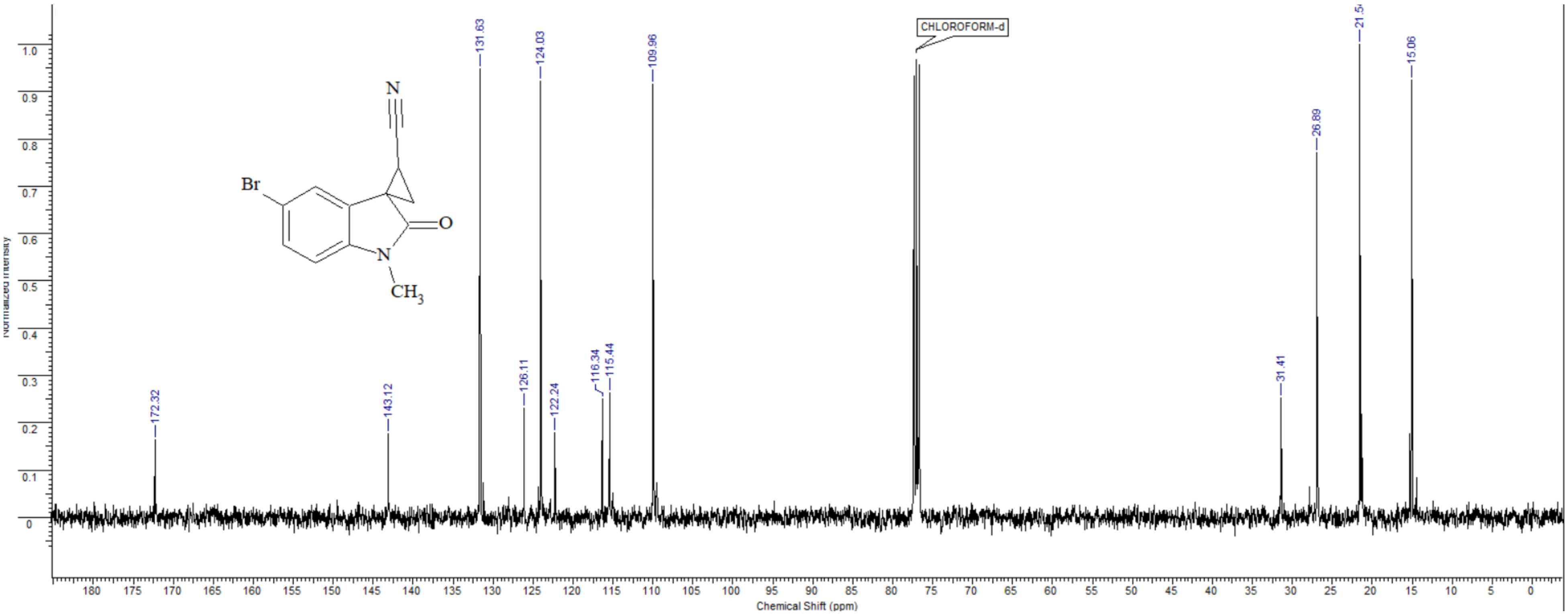

S41 1H NMR spectrum of 1’,6’,7’-Trimethyl-2'-oxo-1',2'-dihydrospiro[cyclopropane-1,3'-indole]-3-carbonitrile (single isomer from 6d) (**6j**)

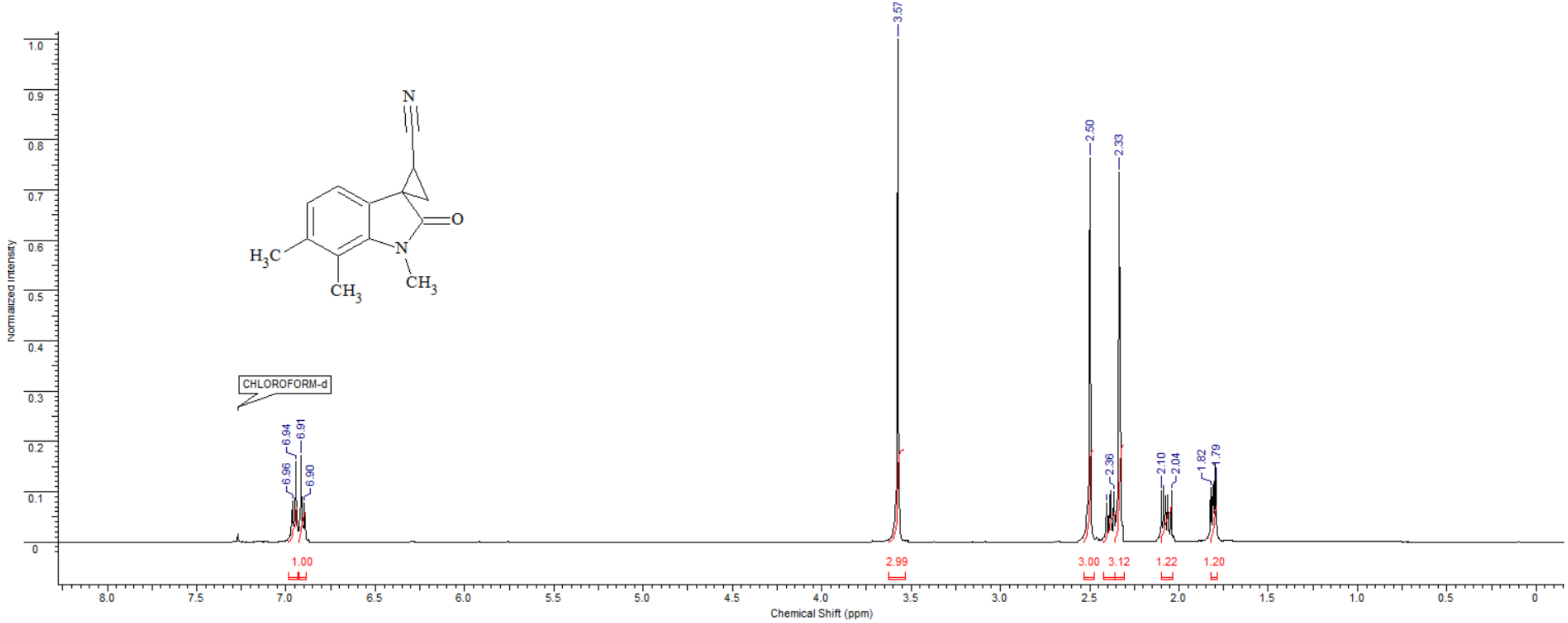

S42 1H NMR spectrum of 1',6',7'-Trimethyl-2'-oxo-1',2'-dihydrospiro[cyclopropane-1,3'-indole]-3-carbonitrile (mixture of isomers from **3j**) (**6j**)

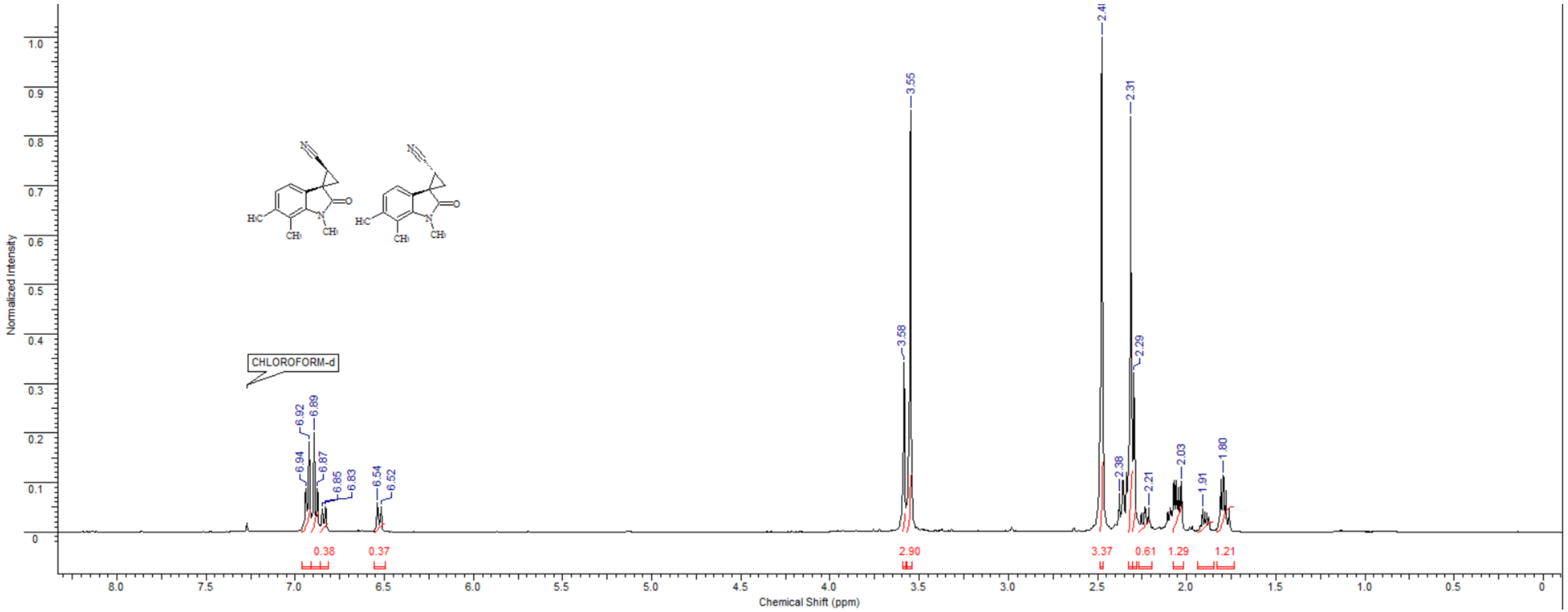

S43 1H NMR spectrum of N-[(1,3-dimethyl-5-methoxy-2-oxo-2,3-dihydro-1H-indol-3-yl)ethyl]acetamide (**7b**)

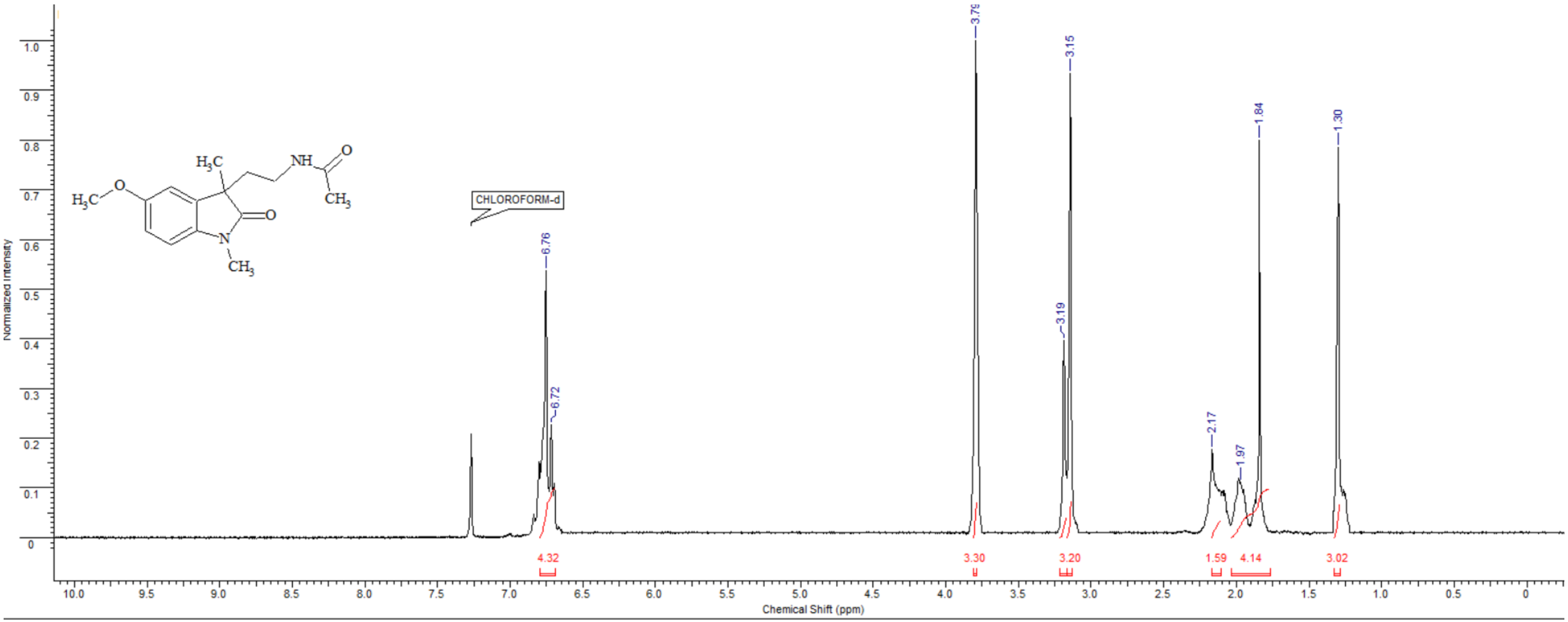

S44 1H NMR spectrum of N-[2-(1,3-Dimethyl-2,3-dihydro-1H-indol-3-yl)ethyl]acetamide (**8a**)

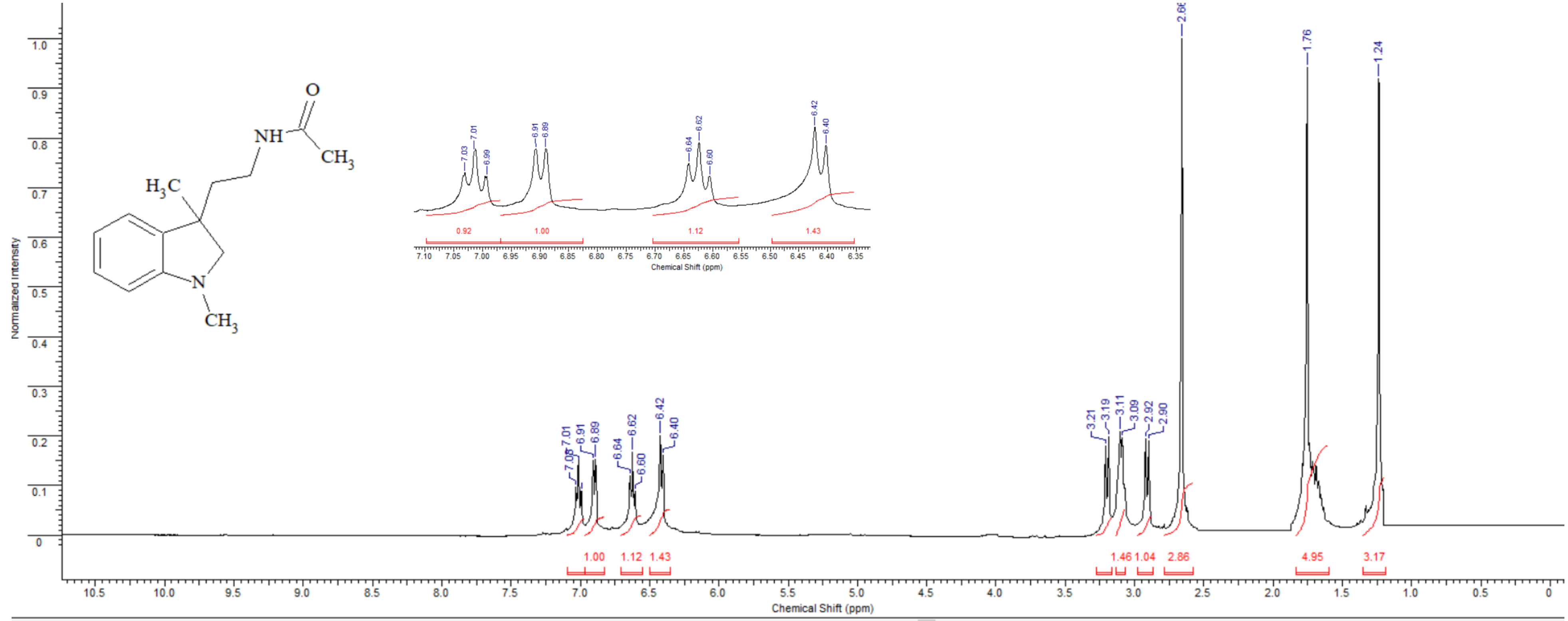

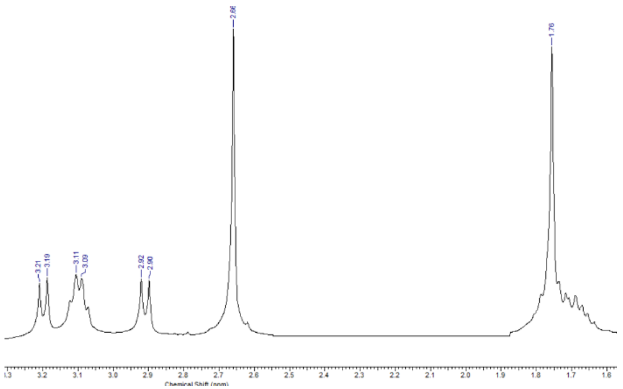

S45 13C NMR spectrum of N-[2-(1,3-Dimethyl-2,3-dihydro-1H-indol-3-yl)ethyl]acetamide (**8a**)

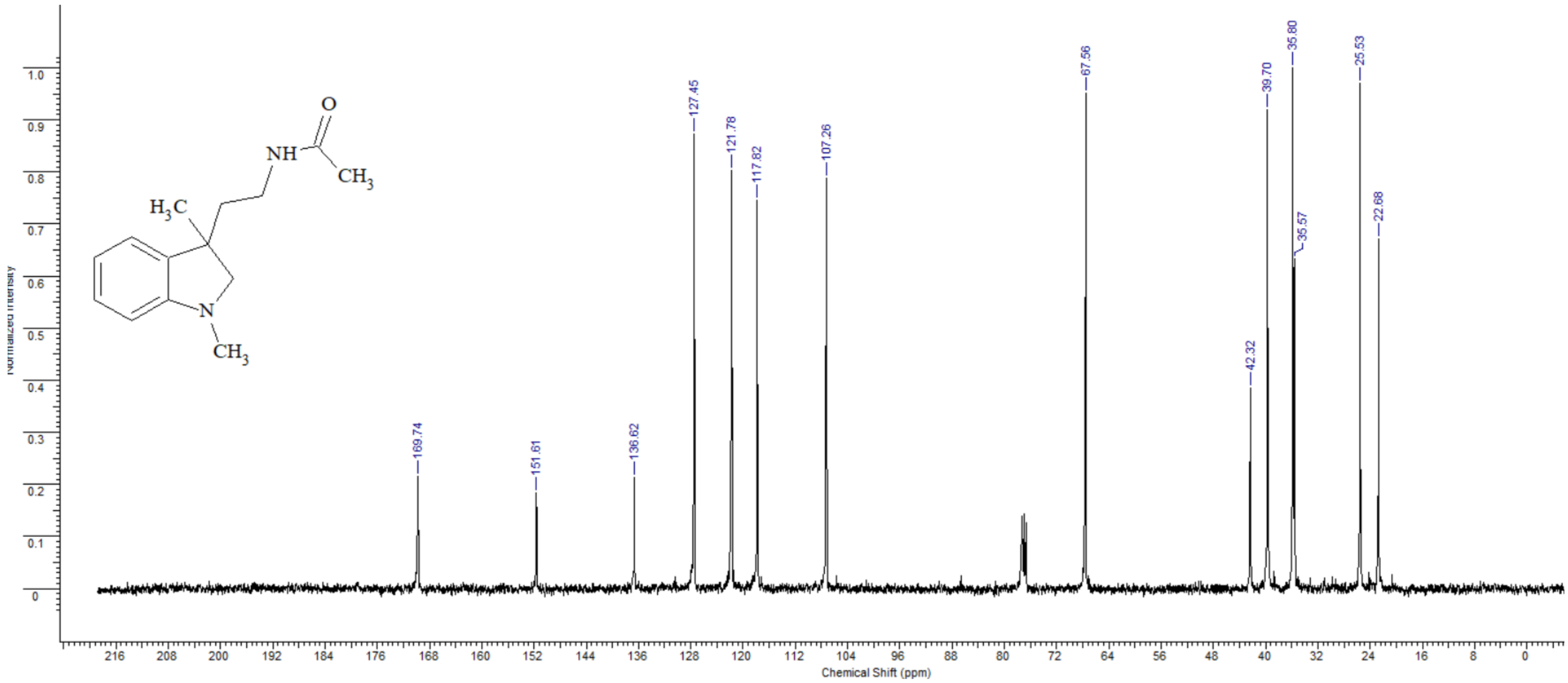

S46 1H NMR spectrum of N-[2-(1-Benzyl-3-methyl-2,3-dihydro-1H-indol-3-yl)ethyl]acetamide (**8b**)

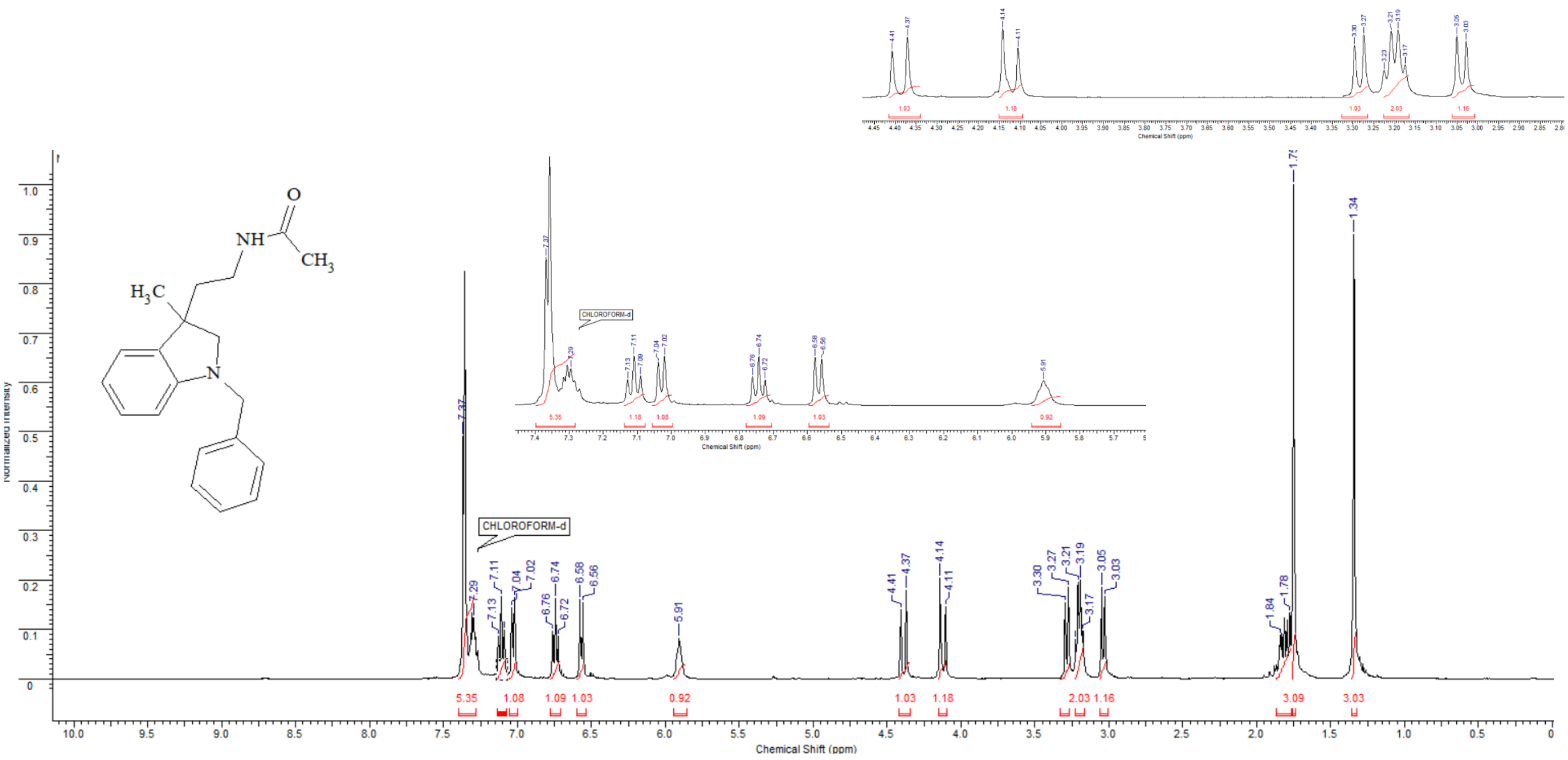

S47 13C NMR spectrum of N-[2-(1-Benzyl-3-methyl-2,3-dihydro-1H-indol-3-yl)ethyl]acetamide (**8b**)

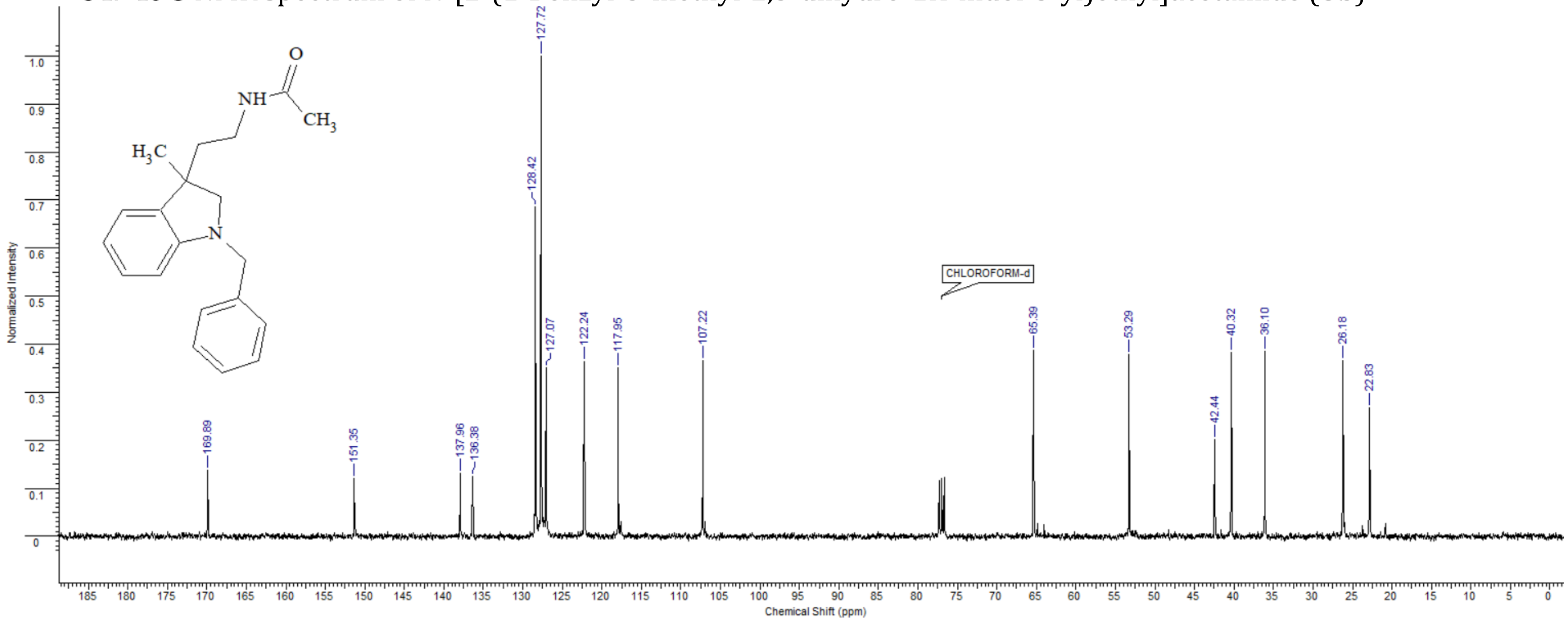

S48 1H NMR spectrum of N-[2-(1,3-Dimethyl-5-methoxy-2,3-dihydro-1H-indol-3-yl)ethyl]acetamide (**8e**)

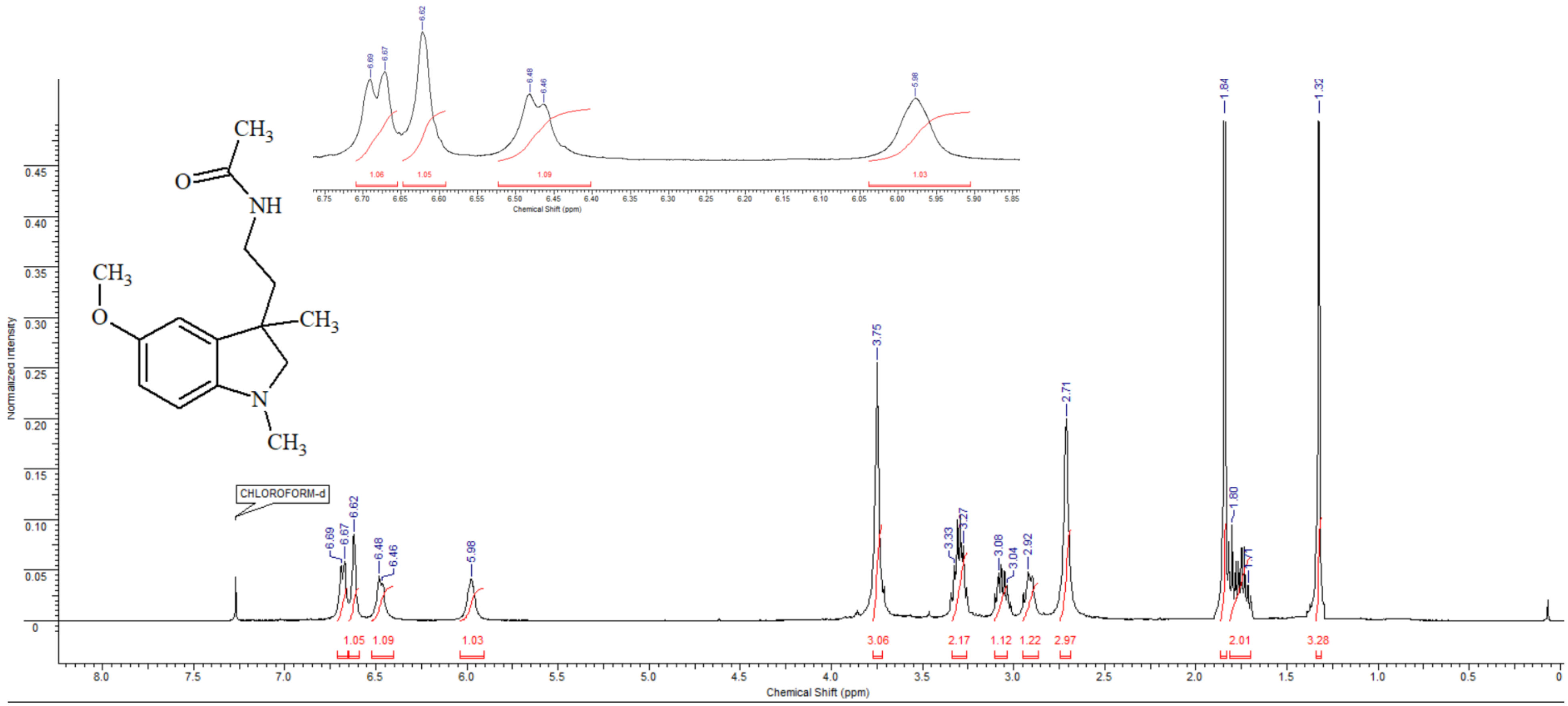

S49 13C NMR spectrum of N-[2-(1,3-Dimethyl-5-methoxy-2,3-dihydro-1H-indol-3-yl)ethyl]acetamide (**8e**)

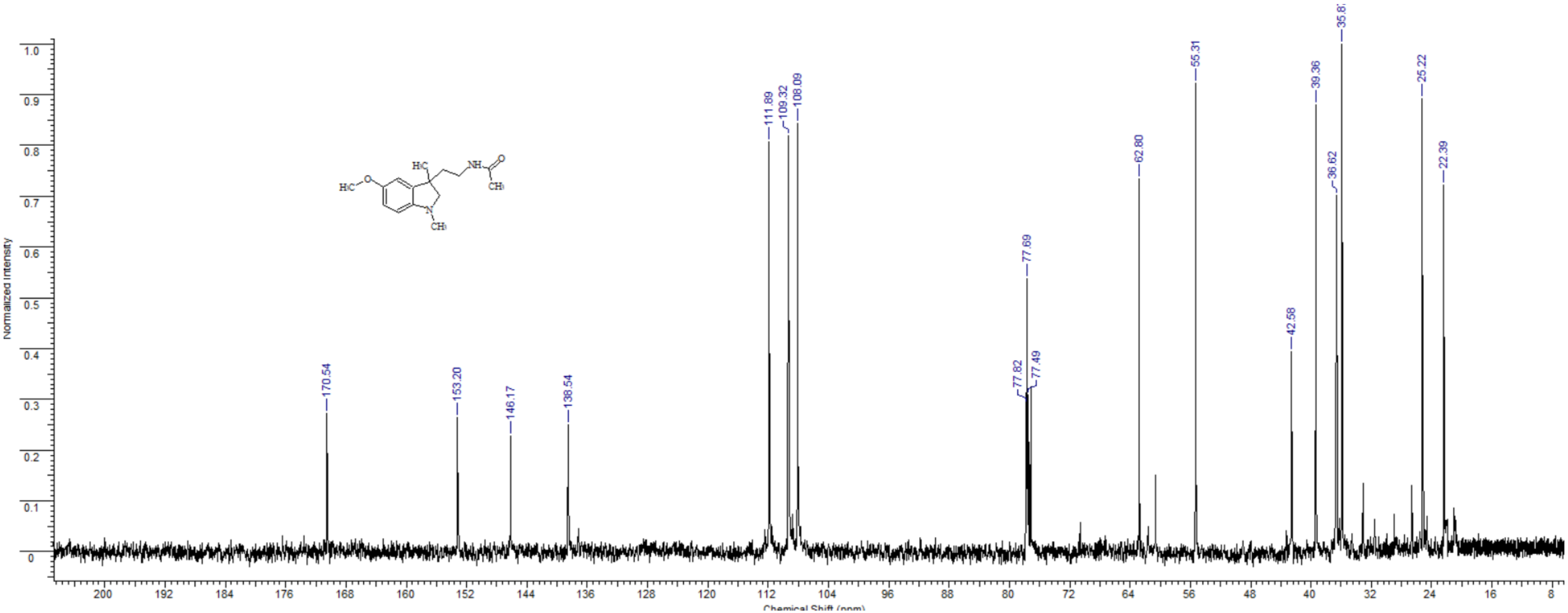

S50 1H NMR spectrum of *N*-[2-(1-Benzyl-3-methyl-5-methoxy-2,3-dihydro-*1H*-indol-3-yl)ethyl]acetamide (**8f**)

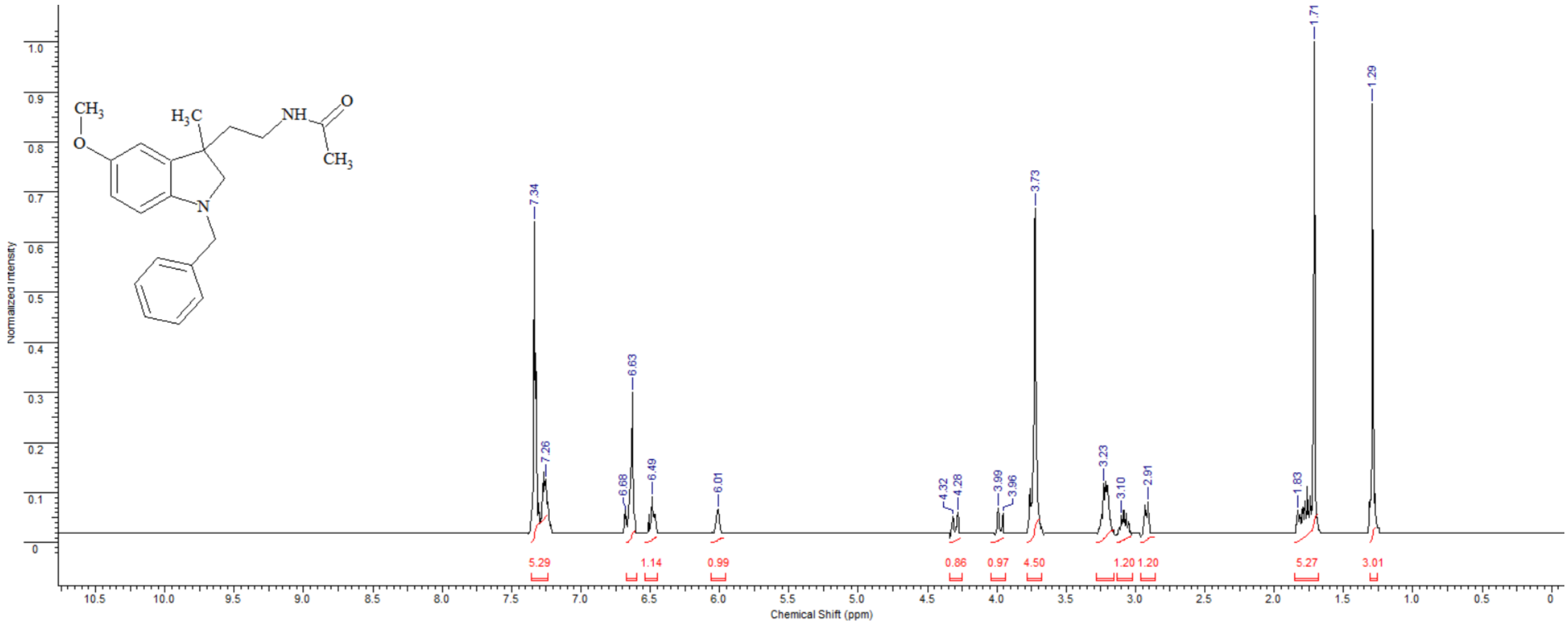

S51 13C NMR spectrum of *N*-[2-(1-Benzyl-3-methyl-5-methoxy-2,3-dihydro-*1H*-indol-3-yl)ethyl]acetamide (**8f**)

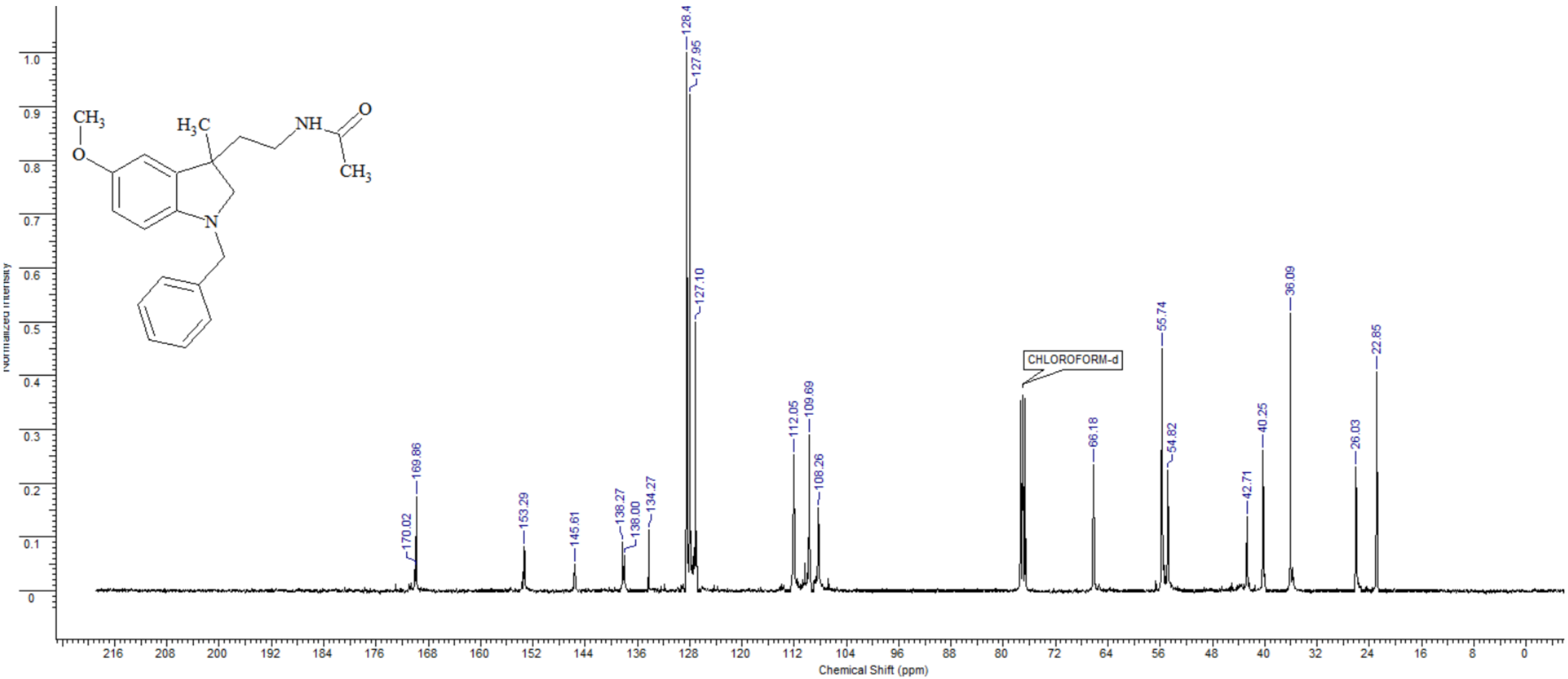

S52 1H NMR spectrum of *N*-[2-(1-Methyl-*1H*-indol-3-yl)ethyl]acetamide (**9i**)

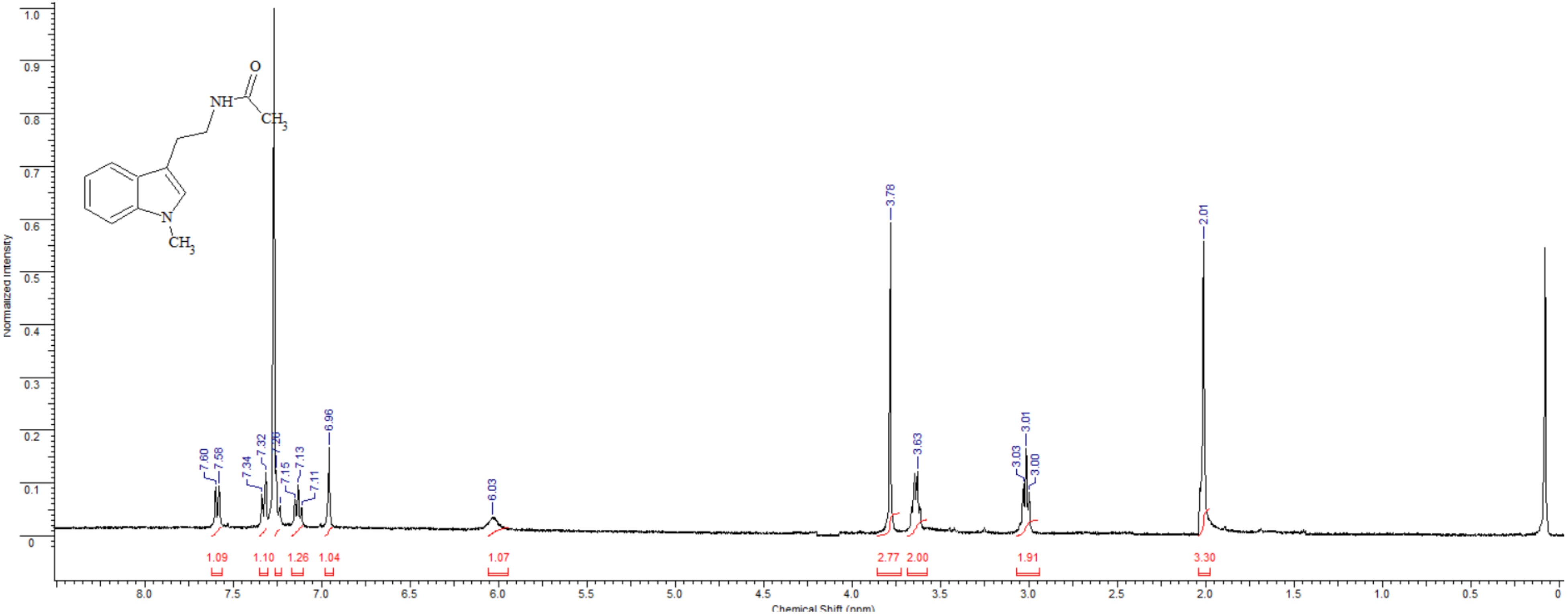

S53 1H NMR spectrum of N-[2-(1,3,6,7-Tetramethyl-2,3-dihydro-1H-indol-3-yl)ethyl]acetamide (**8k**)

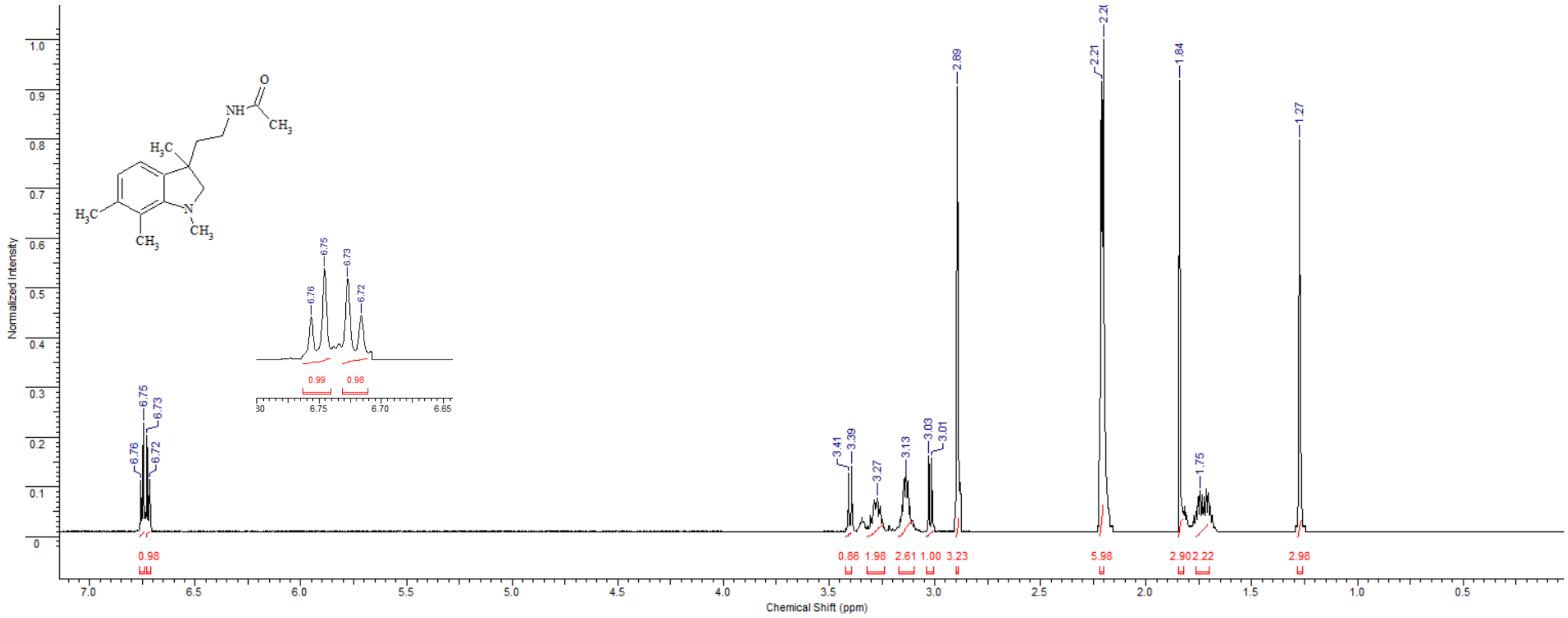

S54 1H NMR spectrum of *N*-[2-(1,6,7-Trimethyl-*1H*-indol-3-yl)ethyl]acetamide (**9l**)

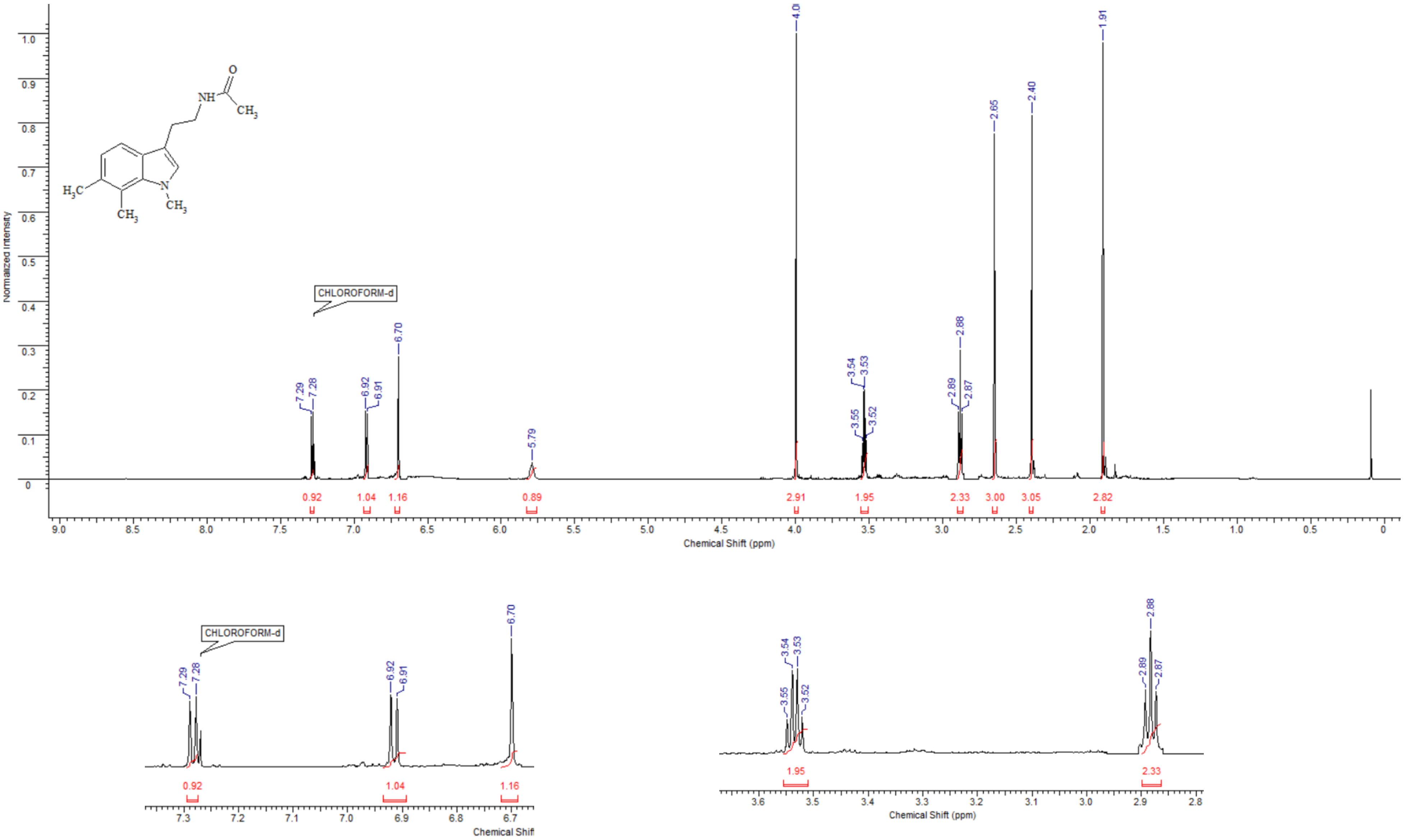

S55 13C NMR spectrum of *N*-[2-(1,6,7-Trimethyl-*1H*-indol-3-yl)ethyl]acetamide (**9l**)

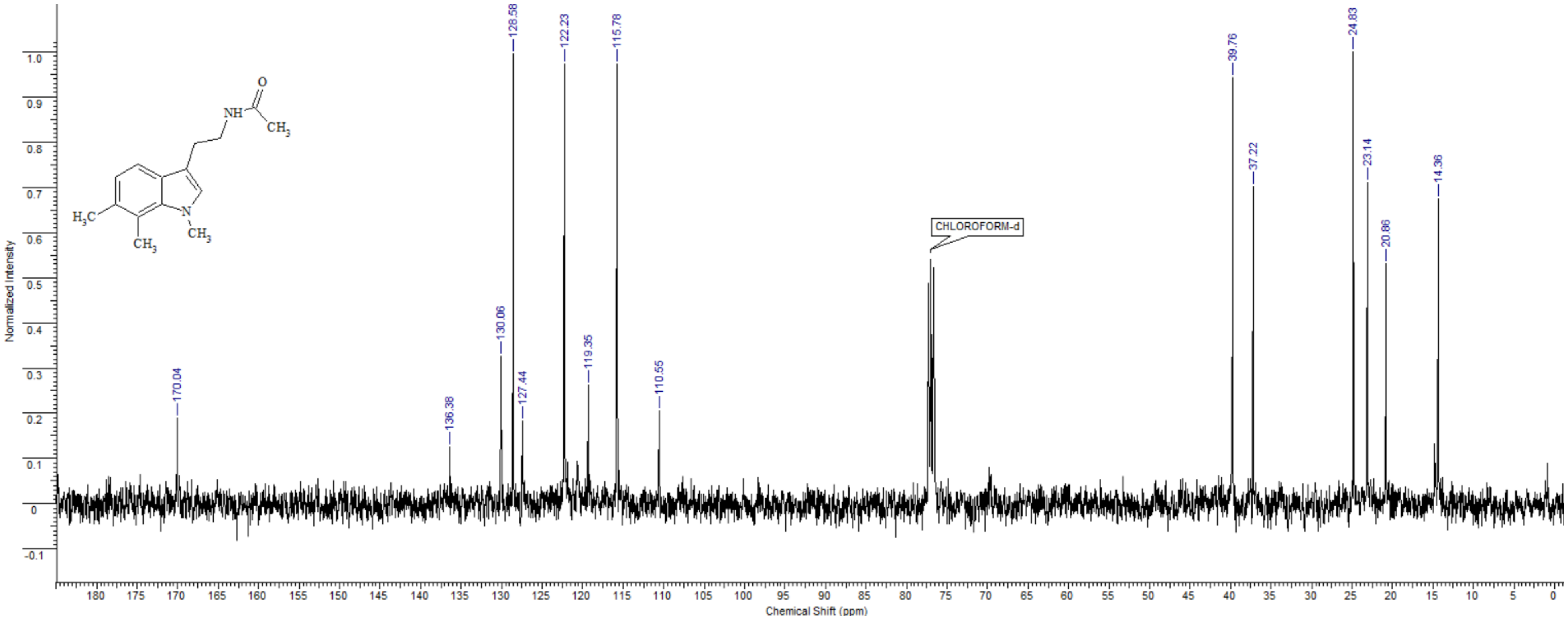

S56 1H NMR spectrum of *N*-[2-(1-benzyl-2,3-dihydro-*1H*-indol-3-yl)ethyl]acetamide (**8m**) and indol (**9m**) after incubation **8m** in DMSO for 1 day.

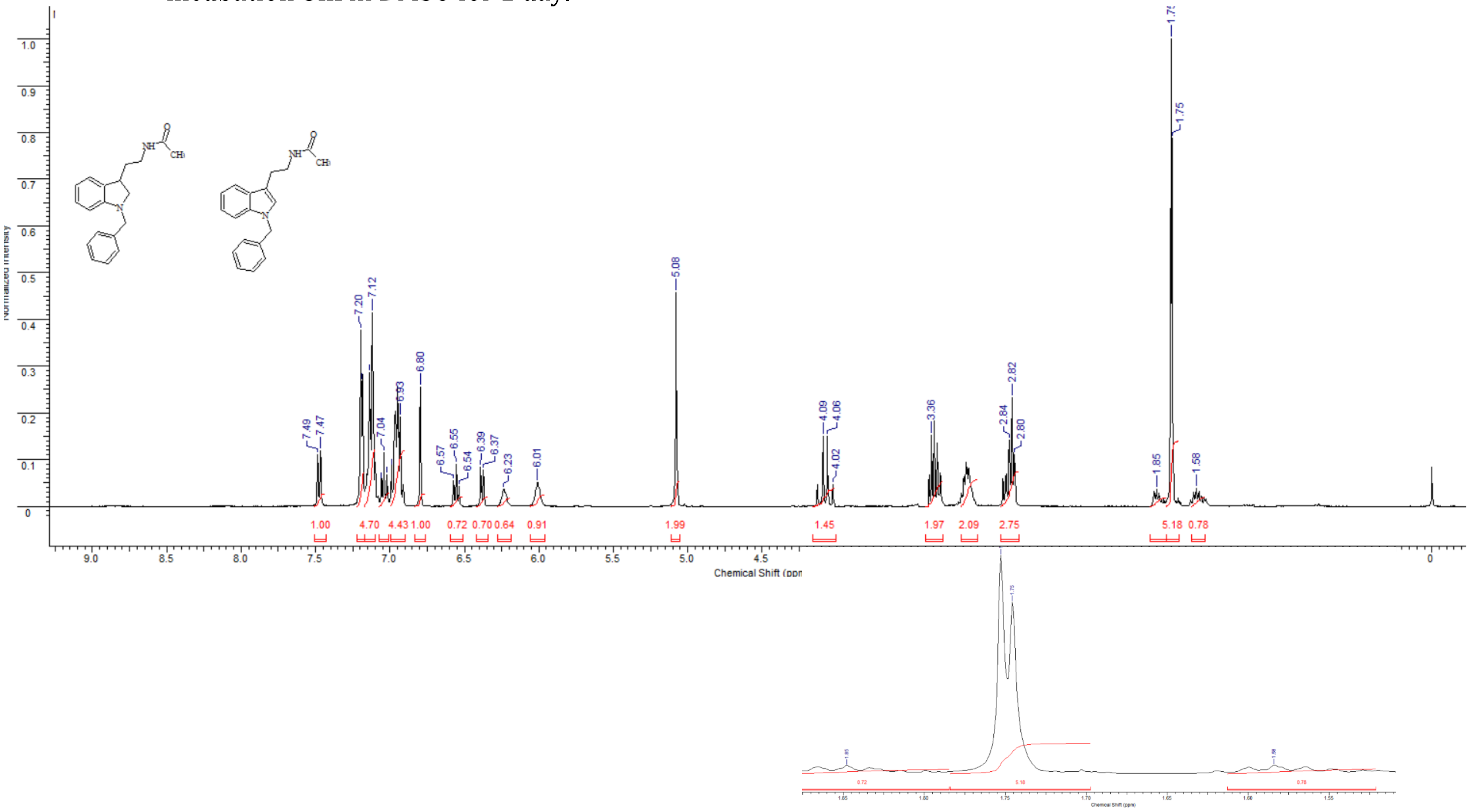

S57 1H NMR spectrum of *N*-{1'-Methyl-1',2'-dihydrospiro[cyclopropan-1,3'-indol]-3-ylmethyl}acetamide (**10a**)

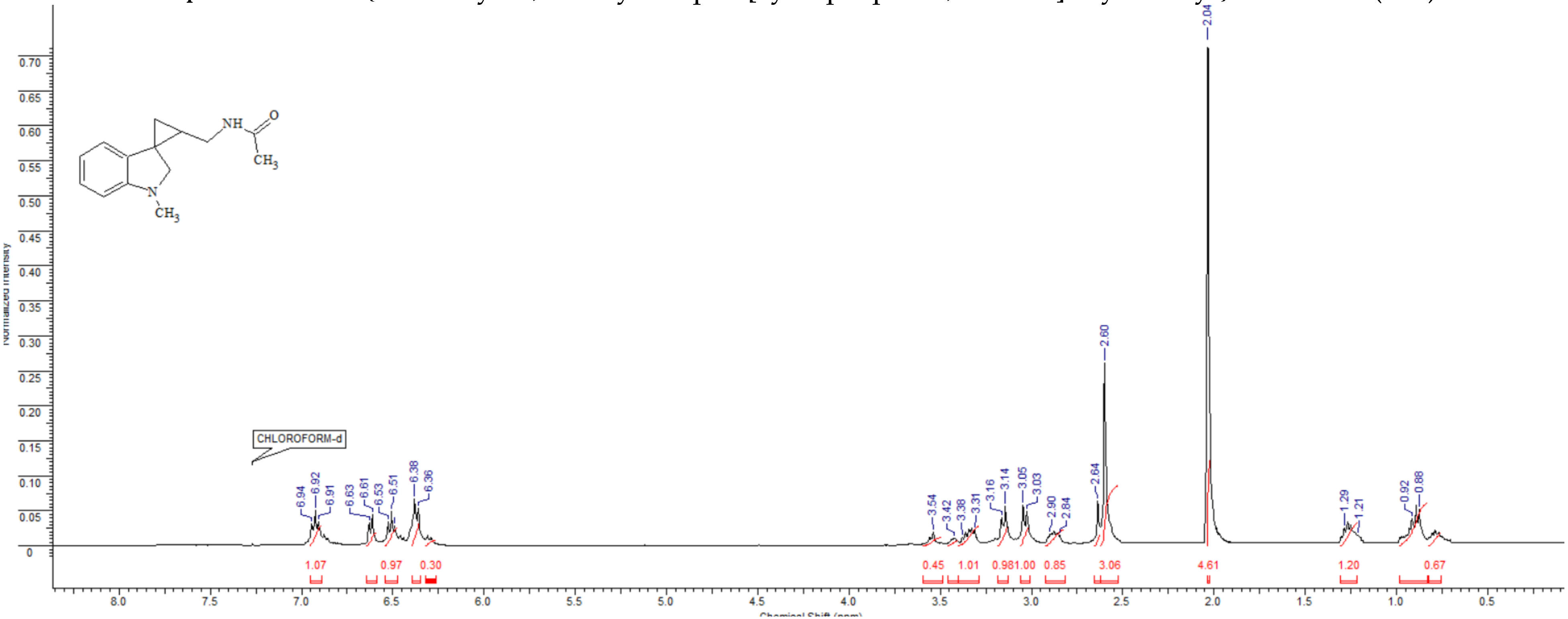

S58 13C NMR spectrum of *N*-{1'-Methyl-1',2'-dihydrospiro[cyclopropan-1,3'-indol]-3-ylmethyl}acetamide (two isomers) (**10a**)

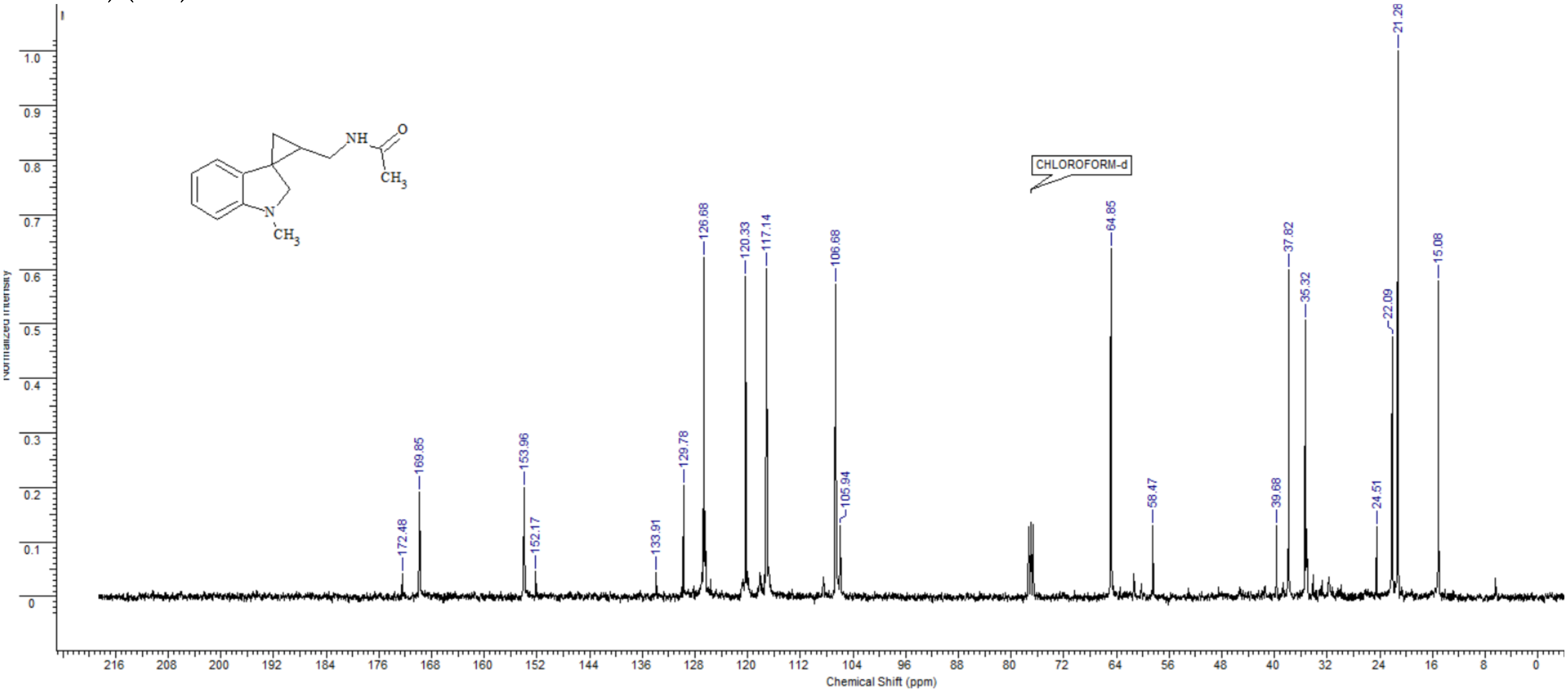

S59 1H NMR spectrum of *N*-{5'-Bromo-1'-methyl-1',2'-dihydrospiro[cyclopropan-1,3'-indol]-3-ylmethyl}acetamide (**10b**)

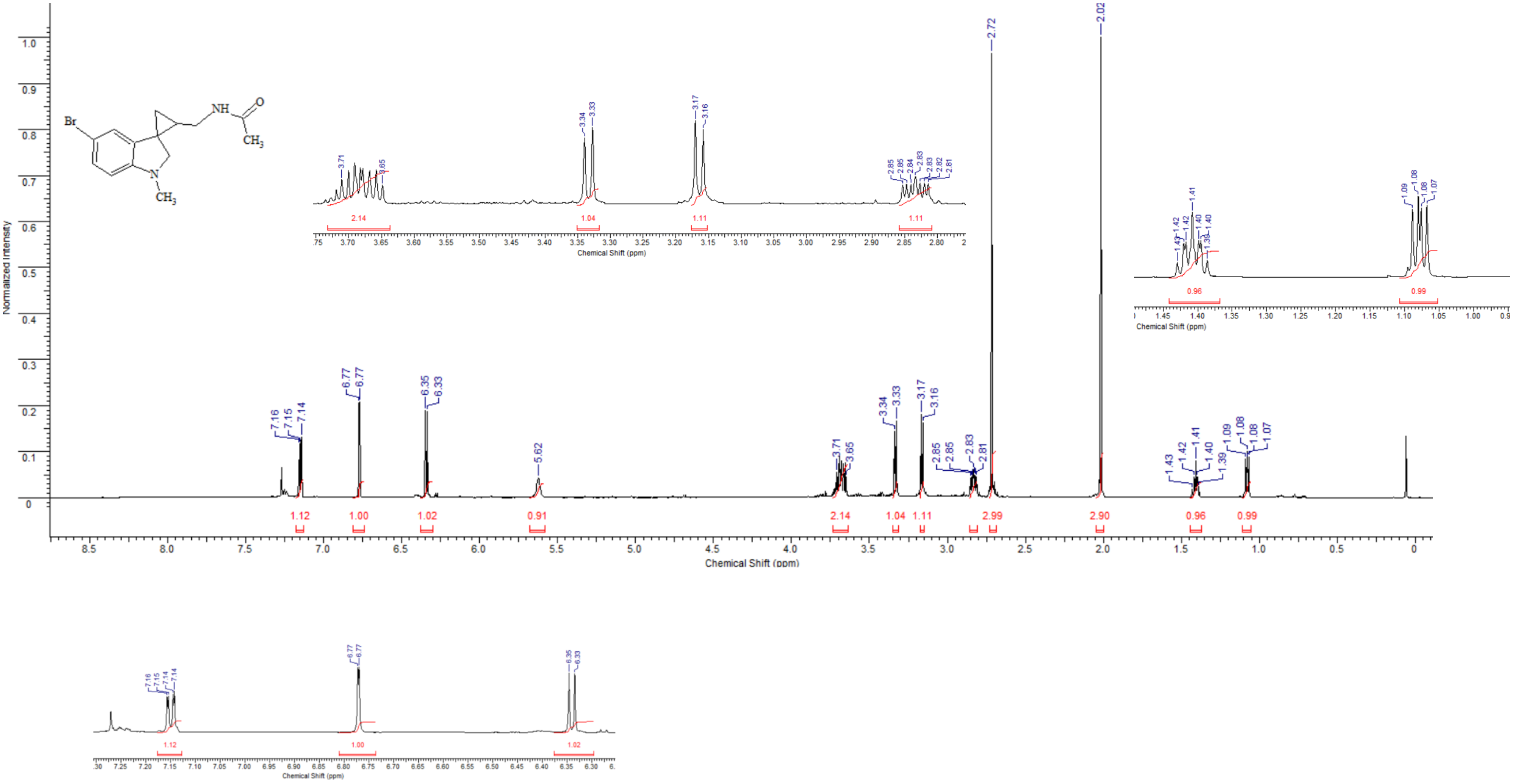

S60 13C NMR spectrum of *N*-{5'-Bromo-1'-methyl-1',2'-dihydrospiro[cyclopropan-1,3'-indol]-3-ylmethyl}acetamide (**10b**)

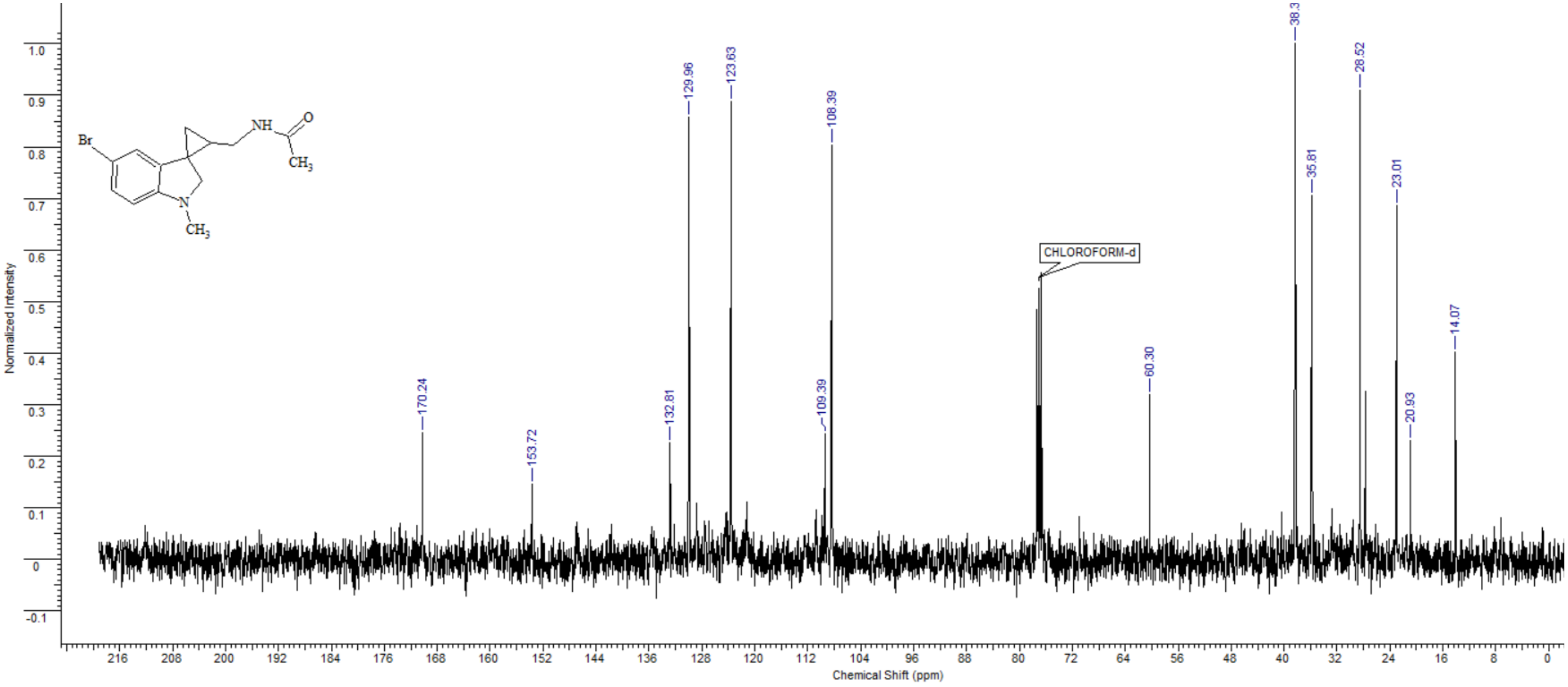

Supplement: Supplementary file 1 [file molecules-27-07462-s001.zip › molecules-1981305-supplementary.pdf]
